# Parabrachial-to-parasubthalamic nucleus pathway mediates fear-induced suppression of feeding in male mice

**DOI:** 10.1038/s41467-022-35634-2

**Published:** 2022-12-30

**Authors:** Takashi Nagashima, Suguru Tohyama, Kaori Mikami, Masashi Nagase, Mieko Morishima, Atsushi Kasai, Hitoshi Hashimoto, Ayako M. Watabe

**Affiliations:** 1 Institute of Clinical Medicine and Research, Research Center for Medical Sciences, The Jikei University School of Medicine, Chiba, Japan; 2 Laboratory of Molecular Neuropharmacology, Graduate School of Pharmaceutical Sciences, Osaka University, Osaka, 565-0871 Japan; 3 Molecular Research Center for Children’s Mental Development, United Graduate School of Child Development, Osaka University, Kanazawa University, Hamamatsu University School of Medicine, Chiba University, and University of Fukui, Osaka, 565-0871 Japan; 4 Division of Bioscience, Institute for Datability Science, Osaka University, Osaka, 565-0871 Japan; 5 Transdimensional Life Imaging Division, Institute for Open and Transdisciplinary Research Initiatives, Osaka University, Osaka, 565-0871 Japan; 6 Department of Molecular Pharmaceutical Sciences, Graduate School of Medicine, Osaka University, Osaka, 565-0871 Japan

**Keywords:** Emotion, Feeding behaviour

## Abstract

Feeding behavior is adaptively regulated by external and internal environment, such that feeding is suppressed when animals experience pain, sickness, or fear. While the lateral parabrachial nucleus (lPB) plays key roles in nociception and stress, neuronal pathways involved in feeding suppression induced by fear are not fully explored. Here, we investigate the parasubthalamic nucleus (PSTN), located in the lateral hypothalamus and critically involved in feeding behaviors, as a target of lPB projection neurons. Optogenetic activation of lPB-PSTN terminals in male mice promote avoidance behaviors, aversive learning, and suppressed feeding. Inactivation of the PSTN and lPB-PSTN pathway reduces fear-induced feeding suppression. Activation of PSTN neurons expressing pituitary adenylate cyclase-activating polypeptide (PACAP), a neuropeptide enriched in the PSTN, is sufficient for inducing avoidance behaviors and feeding suppression. Blockade of PACAP receptors impaires aversive learning induced by lPB-PSTN photomanipulation. These findings indicate that lPB-PSTN pathway plays a pivotal role in fear-induced feeding suppression.

## Abstract (web summary)

Feeding and stress are deeply related to each other, yet neural circuits how they interact are not fully understood. Here, the authors show that the parabrachial-to-parasubthalamic nucleus pathway is involved in fear-induced feeding suppression.

## Introduction

Avoiding potential harm and pursuing nutritious food are critical for animal survival. Accordingly, feeding behavior is deeply related to anxiety and fear. Feeding suppression occurs when animals experience a wide range of stress, including gastrointestinal malaise, predator odors, chronic restraint stress, acute pain, and fear memory^1–6^. In pain signal processing and fear memory formation, the lateral parabrachial nucleus (lPB) plays a crucial role^7,8^. Specifically, the lPB is activated by nociceptive signals and transmits to brain regions critical for defensive behaviors and aversive signal perception^9–17^. Recent reports demonstrated that the lPB plays a key role in fear-induced suppression of feeding^2^. Activation of lPB neurons projecting to the central amygdala (CeA) or lateral hypothalamus (LH) induces feeding suppression^18,19^. These lines of evidence suggest that the lPB is well situated to serve as a hub for stress signals and feeding behaviors. Although neural circuits for feeding have been extensively studied in the hypothalamus^20,21^, neural circuits controlling feeding suppression induced by fear and pain are still not fully explored.

Pituitary adenylate cyclase-activating polypeptide (PACAP) is a well-conserved neuropeptide that plays pivotal roles in chronic pain, anxiety, and stress-related psychopathologies^22–29^. Furthermore, a growing body of evidence demonstrates that PACAP is also involved in feeding suppression^6,30^. Here, we detected the parasubthalamic nucleus (PSTN) as a downstream target of the lPB as previously reported^5^. We found that PACAP is highly enriched in the PSTN.

The PSTN is located in the lateral subdivision of the posterior hypothalamus^31^. The PSTN recently emerged as a critical interface between interoception and emotions^32^, and for the regulation of appetite suppression^33^. Yet, its physiological roles in health and disease are still largely unexplored. Therefore, our observations led us to hypothesize that the lPB-PSTN pathway could be involved in fear-induced feeding suppression via PACAP signaling. To directly address this issue, we investigated the role of lPB-PSTN in aversive behaviors and feeding regulation using optogenetics, pharmacogenetics, electrophysiology, and pharmacological analysis. We propose that the lPB-PSTN pathway contributes to the fear-induced suppression of feeding behaviors.

## Results

### Histological and electrophysiological characterizations of the lPB-PSTN pathway

**Fig. 1:**
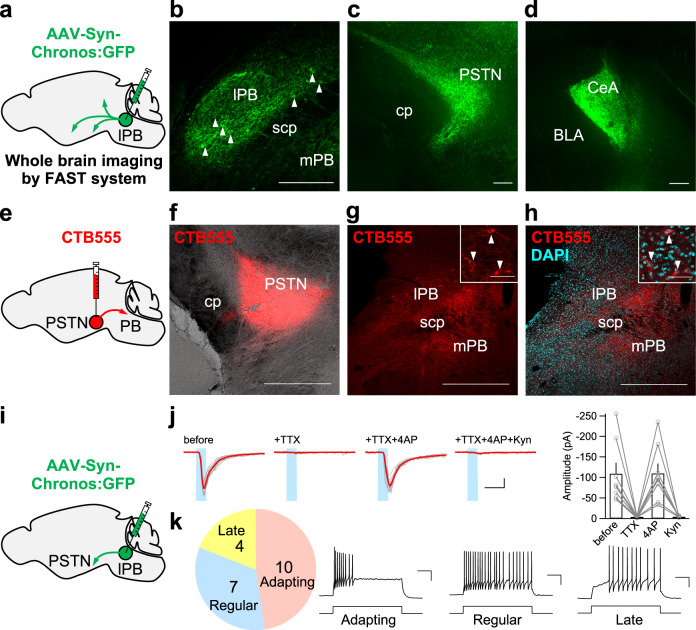
Mapping of the projection of lateral PB neurons and characterizations of the lPB-PSTN pathway. **a** Schematic of microinjection into the lateral PB (lPB) of the C57BL/6J mice. **b** Images of fluorescence at the injection site, lPB. Arrowheads point to the cell bodies of Chronos:GFP-expressing cells. scp, superior cerebellar peduncle. **c**, **d** Images of fluorescence at the projection sites of lPB neurons, PSTN (**c**) and CeA (**d**). cp, cerebral peduncle. Scale bars represent 200 μm. Experiments were repeated independently in at least two mice with similar results. **e**, **f** Schematic illustration and representative image of microinjection of CTB555, a retrograde tracer, into the PSTN of C57BL/6J mice. Scale bar represents 500 μm. **g**, **h** Representative image showing the retrogradely labeled PB neurons (**g**), and image merged with DAPI (**h**). Scale bars represent 50 μm (inset) or 500  μm. Experiments were repeated independently in at least three mice with similar results. **i** Bilateral injection of AAV-Syn-Chronos:GFP into lPB of C57BL/6J mice. **j** Traces of EPSCs (gray, 15 consecutive responses; red, average) evoked by photo-stimulation (every 20 s, 5-ms duration). Data are represented as mean + SEM. Scale bar, 50 pA and 10 ms. Right panel represents summary of the EPSC amplitudes (*n* = 8 cells). **k** Firing patterns of PSTN neurons (*n* = 21 cells). Scale bar, 20 mV and 200 ms. See also Supplementary Fig. 1.

We first confirmed the projection sites of lPB neurons. Following injection of AAV-Syn-Chronos:GFP into the bilateral lPBs of adult mice, projection sites were examined block-face serial microscopy tomography system (FAST) (Fig. 1a)^34,35^. Fluorescence was observed in the injection site (Fig. 1b; Supplementary Movie 1), and projection sites such as the PSTN, CeA, periaqueductal gray (PAG), ventral tegmental area (VTA), ventral posteromedial thalamic nucleus (VPMpc), ventromedial hypothalamus (VMH), insular cortex (IC), and bed nucleus of stria terminalis (BNST) (Fig. 1c, d and Supplementary Fig. 1). Because the PSTN showed strong fluorescence, we next investigated the histological and electrophysiological properties of the PSTN-projecting lPB neurons.

Retrograde tracer cholera toxin subunit B (CTB) was injected into the PSTN (Fig. 1e, f). PSTN-projecting PB neurons were primarily located in the lPB including the dorsal and external lPB (Fig. 1g, h). These results are consistent with the previous studies for the existence of the lateral hypothalamus-projecting neurons in the lPB^5,19^.

To characterize the physiological properties of the lPB-PSTN pathway, we next performed electrophysiological analyses using the whole-cell patch-clamp recordings. Briefly, following injection of AAV-Syn-Chronos:GFP into the lPB of mice, light-evoked excitatory postsynaptic currents (EPSCs) were recorded from visually identified PSTN neurons (Fig. 1i). We detected EPSCs evoked by a 5-ms light pulse in voltage-clamp modes at –60 mV (Fig. 1j). These EPSCs were blocked by the voltage-dependent sodium channel blocker, tetrodotoxin (TTX; 1 μM). The EPSCs were recovered by the addition of 100 μM 4-aminopyridine (4-AP), while subsequent application of kynurenic acid (Kyn; 3 mM) abolished EPSCs. We also analyzed the firing patterns of PSTN neurons and categorized them into three different types; adapting, regular-spiking, and late-spiking. The mean light-evoked EPSC amplitude was similar among them. We found that the PSTN contains a mixture of different types of neurons, and the lPB neurons project to multiple types of neurons (Fig. 1k). These results indicate that lPB-PSTN projections are monosynaptic and glutamatergic pathway. We therefore next investigated the function of the lPB-PSTN pathway in behavioral regulation.

### Activation of the lPB-PSTN pathway promotes real-time place avoidance as well as fear memory formation

**Fig. 2:**
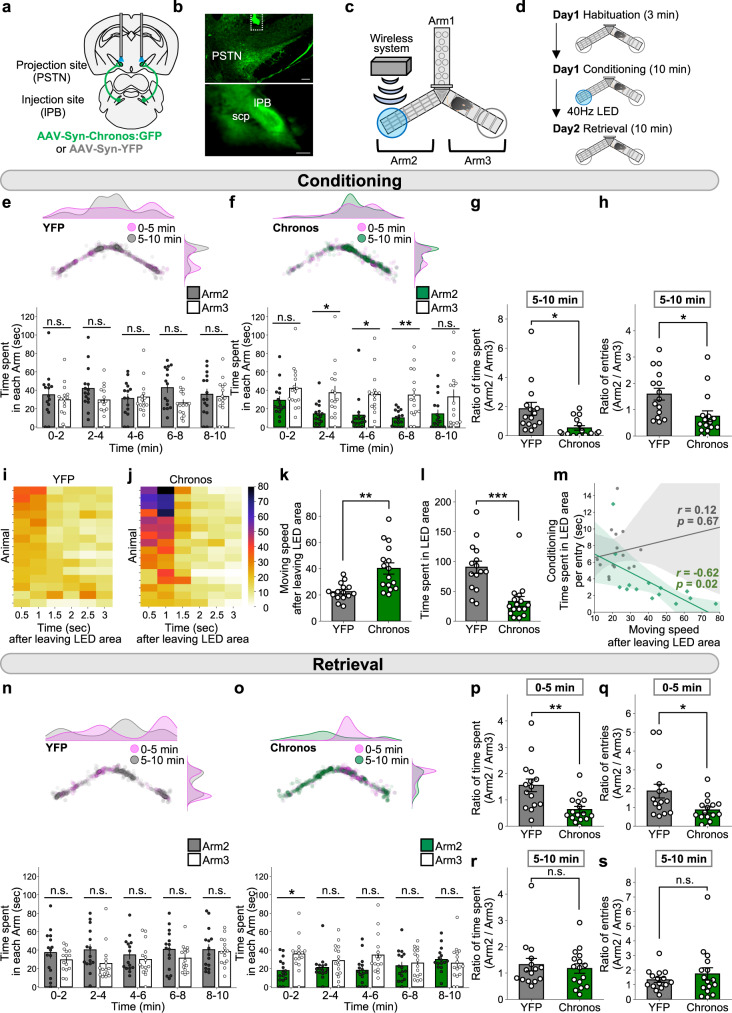
Photoactivation of the lPB-PSTN promotes avoidance behavior and aversive learning. **a** Schematic of microinjection and placement of the LED cannula unit of the C57BL/6J mouse. **b** Images of the projection site, in the PSTN (top) and the injection site in the lPB (bottom). The dashed line in the top image indicates the position of the optic fiber. Scale bars, 200 μm. scp, superior cerebellar peduncle. More than five independent experiments were conducted and similar results were obtained. **c** Schematic illustrations of the Y-shaped apparatus. **d** Experimental schedule of the place avoidance test. **e**, **f** Density plots and a scatter plot showing the position of a representative YFP (**e**) and Chronos (**f**) mouse every 0.5 s in the 10-min conditioning session (top). Time spent in each arm during each 2-min interval (bottom) (YFP, *n* = 15; Chronos, *n* = 16). **g**, **h** Summary of ratios of time spent in and entries into Arm 2/Arm 3 for 5–10 min of the conditioning session (YFP, *n* = 15; Chronos, *n* = 16). **i**, **j** Heatmaps represent averaged moving speed of each mouse during the 10-min conditioning session. **k** Average speed for a second after mice left the LED area during the 10-min conditioning session (YFP, *n* = 15; Chronos, *n* = 16). **l** Time spent in the LED area during the 10-min conditioning session (YFP, *n* = 15; Chronos, *n* = 16). **m** Correlations of time spent in LED area and moving speed after leaving LED area. Gray and green plots indicate YFP and Chronos mice, respectively. Regression lines were drawn with 95% confidence intervals. Pearson’s correlation coefficients were shown (YFP, *r* = 0.12, *p* = 0.67; Chronos, *r* = –0.62, *p* = 0.02). **n**, **o** The position of a mouse in the 10-min retrieval session (top) and the time spent in each arm during each 2-min interval (bottom) (YFP, *n* = 15; Chronos, *n* = 16). **p**–**s** Summary of ratios of time spent in and entries into Arm 2/Arm 3 during the retrieval session (YFP, *n* = 15; Chronos, *n* = 16). Data are represented as mean ± SEM (**e**–**h**, k, **l**, and **n**–**s**). n.s., *p* > 0.05; *p < 0.05; **p < 0.01; and ***p < 0.001 (two-way repeated measures ANOVA followed by Bonferroni post hoc test (**e**, **f**, **n**, and **o**) and unpaired two-sided *t*-test (**g**, **h**, **k**, **l**, and **p**–**s**)). See also Supplementary Fig. 2 and Supplementary Movie 2.

To this end, we assessed real-time place avoidance behavior using a Y-shaped apparatus with a wireless photostimulation system. After bilateral AAV injection (AAV-Syn-Chronos:GFP or AAV-Syn-YFP) into the lPB, dual optic cannulae were implanted over the PSTN for optogenetic activation of axonal terminals (Fig. 2a, b), as previously reported^11^. The placement of the LED cannula is shown in Supplementary Fig. 2a. Although it is possible that stimulation of a single projection target may result in antidromic activation of other target regions, recent papers have suggested it is not highly likely in projections from lPB neurons^36,37^.

More than 2 h before the conditioning session (Day 1), mice were habituated to the Y-maze apparatus for 3 min (Fig. 2d). Neither the ratio of time spent in Arm 2 to Arm 3, nor the ratio of number of entries into Arm 2 to Arm 3 was significantly different between YFP control and Chronos mice (Supplementary Fig. 2b–d). These data suggest that mice have no bias in texture or right-left preference in the habituation session.

In the conditioning session, mice freely explored the Y-maze apparatus between Arm 2 and Arm 3, and received photostimulation (5 ms, 40 Hz) whenever they entered the LED area set on the distal half of Arm 2 (Fig. 2c). We first examined general locomotion in the conditioning session. Total traveling distances were not significantly different between control and Chronos mice (Supplementary Fig. 2e), suggesting that general locomotor activity was not impaired by photostimulation. Although control mice showed no biases between the two arms (Fig. 2e and Supplementary Movie 2), Chronos mice began to avoid Arm 2 (including the LED area) from two minutes after the start of the conditioning session (Fig. 2f and Supplementary Movie 2). As a result, the ratio of time spent (Arm 2/Arm 3) and the time spent in the LED area per entry from 5–10 min were significantly shorter in Chronos mice (Fig. 2g and Supplementary Fig. 2f). These findings indicate that activation of the lPB-PSTN pathway drives avoidance behavior during the conditioning session. The ratio of entries (Arm 2/Arm 3) was also decreased in Chronos mice (Fig. 2h), suggesting that activation of the lPB-PSTN pathway also alters a behavioral choice.

During the conditioning session, Chronos mice quickly ran out of the LED area. Moving speed for 3 s after leaving the LED area was analyzed for 0.5-s intervals and visualized as a heatmap, in which the darkness of color indicates more rapid movement (Fig. 2i, j). Moving speeds after leaving the LED area were significantly larger in Chronos mice compared with control mice (Fig. 2k). Moreover, total time spent in the LED area was decreased in Chronos mice (Fig. 2l). These results suggest that photoactivation of the lPB-PSTN pathway immediately induces escape behavior. We also found that the observed escape behavior correlated with avoidance behavior. Specifically, Chronos mice showed a positive correlation between their moving speed during the first second after leaving from the LED area and the time spent in the LED area per entry from 0–5 min (Pearson correlation coefficient: YFP, *r* = 0.12, *p* = 0.67; Chronos, *r* = –0.62, *p* = 0.02; Fig. 2m). These results indicate that photoactivation of the lPB-PSTN pathway in mice causes escape behavior and bias toward the arm not containing the LED area.

The day after the conditioning session, a memory retrieval test was performed in which mice were placed in the same apparatus used for the conditioning session but without photostimulation. Chronos mice avoided Arm 2 for 6 min after the start of the retrieval session, while the control mice did not (Fig. 2n, o). Ratios of time spent and entry in each arm (Arm 2/Arm 3) from 0–5 min were significantly shorter in Chronos mice (Fig. 2p, q). In addition, time spent in the area equivalent to the LED area per entry tended to be decreased in Chronos mice from 0–5 min (Supplementary Fig. 2g). These results indicate that activation of the lPB-PSTN pathway established a robust memory. However, from 5–10 min, neither the ratio of time spent nor the ratio of entry into each arm was changed (Fig. 2r, s), suggesting that memory extinction may have occurred during the latter half of the retrieval session.

We also conducted the lever-press experiment combined with optogenetic stimulation. Mice were trained to press the lever using a real food pellet, then subjected to the test in which LED illumination (5 ms, ten 20-Hz pulses) was delivered to the lPB-PSTN pathway only when the mice pressed the lever (Supplementary Fig. 3a). Chronos mice exhibited significantly fewer numbers of lever presses compared to those of control mice (Supplementary Fig. 3b, c). These results further support the notion that the lPB-PSTN pathway serves aversive signals.

### Activation of the lPB-PSTN pathway suppresses feeding behaviors

**Fig. 3:**
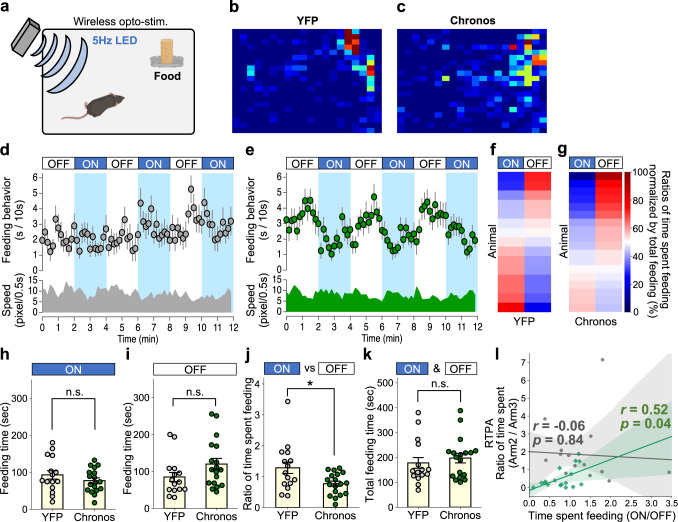
The lPB-PSTN stimulation suppresses feeding. **a** Schematic illustration of the feeding test. **b**, **c** Each heatmap represents time spent in the area for a representative mouse during a total 6 min with photostimulation (2-min photostimulation period repeated three times). **d**, **e** Time course of feeding behavior of YFP (**d**) and Chronos mice (**e**). Data are represented as mean ± SEM (YFP, *n* = 15; Chronos, *n* = 18). Each circle represents average feeding time every 10 s. Average moving speed every 10 s is shown on the bottom. **f**, **g** Heatmaps represent ratios of time spent feeding during LED-on (6 min) and LED-off (6 min) to total feeding time for each individual mouse. **h**, **i** Time spent feeding during LED-on and LED-off periods. **j** Ratios of time spent feeding in LED-on/LED-off periods. **k** Total time spent feeding during the 12-min observation period. Each circle represents results from one mouse (YFP, *n* = 15; Chronos, *n* = 18). Data are represented as mean ± SEM. n.s., *p* > 0.05; *p < 0.05 (Unpaired two-sided *t*-test). **l** Correlations of ratios of time spent feeding and time spent in each arm in the real-time place avoidance (RTPA) test in Fig. 2, which would represent feeding and aversive behavior, respectively. Each dot represents a value obtained from one mouse (gray, YFP; green, Chronos). Regression lines were drawn with 95% confidence intervals. Pearson’s correlation coefficients were shown (YFP, *r* = –0.06, *p* = 0.84; Chronos, *r* = 0.52, *p* = 0.04). See also Supplementary Fig. 4 and Supplementary Movie 3.

The PSTN plays a key role in the termination of feeding behaviors^38,39^. A recent paper demonstrated that the PSTN plays a critical role in appetite suppression^33^. Therefore, we examined whether the lPB-PSTN pathway modulates feeding behavior by optogenetically activating the lPB terminals in the PSTN. The test chamber was identical to a homecage, and a food pellet was fixed on the floor at the corner of the chamber (Fig. 3a). The mice used for the Y-maze experiments were subjected to feeding behavior analysis. First, mice were habituated to the test chamber without photostimulation for 2 d after being food-deprived for 18–22 h. During the test session, photostimulations (5 ms, five 5-Hz pulses for 2 min) were applied to the lPB-PSTN pathway. We confirmed that general locomotor activity was not remarkably changed by photostimulation (Supplementary Fig. 4a). Heatmaps representing the time spent in each region of the test chamber during the total 6-min photo-stimulation period are shown in Fig. 3b, c. The control group did not exhibit a remarkable change in time spent feeding (Fig. 3d). Conversely, Chronos mice stopped feeding immediately when the LED was turned on (LED-on) and started feeding again when it was turned off (LED-off); these effects of the LED were repeatedly observed (Fig. 3e and Supplementary Movie 3). We calculated ratios of time spent feeding during LED-off (6 min) and LED-on (6 min) to total feeding time for each individual mouse (color-coded in Fig. 3f, g). Most Chronos mice exhibited a lower ratio time spent feeding during LED-on (6 min) compared with LED-off (6 min). Feeding times of Chronos and YFP mice were not significantly different but tended to change when the LED was turned on or off (Fig. 3h, i). Specifically, the ratio of time spent feeding was decreased for LED-on/Total and increased for LED-off/Total in Chronos mice (Supplementary Fig. 4b, c). The ratio of time spent feeding for LED-on/LED-off was decreased in Chronos mice compared with control mice (Fig. 3j). In addition, Chronos mice more frequently exhibited food-leaving behavior during LED-on (Supplementary Fig. 4d, e). These results suggest that activation of the lPB-PSTN inhibits feeding behavior. In contrast, total feeding time and total food intake were not significantly changed (Fig. 3k and Supplementary Fig. 3f). These results suggest that the lPB-PSTN pathway transmits aversive information and negatively regulates feeding behavior in a short period of time. We next analyzed the relationship between the real-time place aversion test results (Fig. 2e–m) and feeding test results in individual mice. Comparison of the ratio of time spent in Arm 2/Arm 3 for the place avoidance test with the ratio of time spent feeding for LED-on/LED-off for the feeding test revealed a positive correlation in Chronos mice (Fig. 3l; Pearson correlation coefficient: YFP, *r* = –0.06, *p* = 0.84; Chronos, *r* = 0.52, *p* = 0.04). Taken together, these results support the idea that activation of the lPB-PSTN pathway is sufficient to inhibit feeding behavior.

### Inhibition of PSTN neurons attenuates fear-induced suppression of feeding

**Fig. 4:**
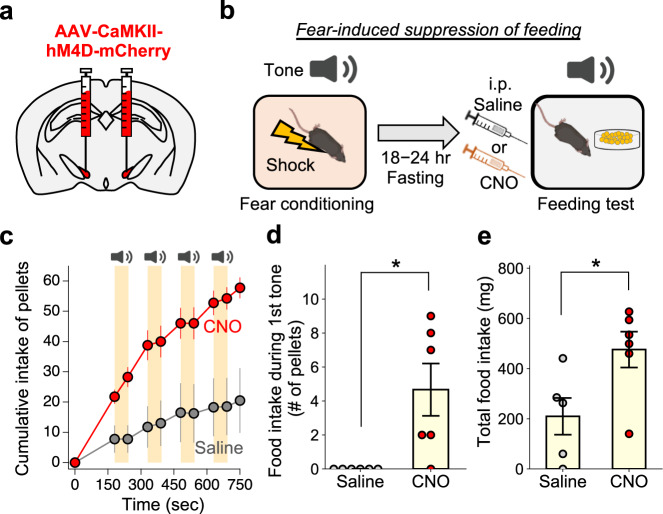
Inhibition of PSTN neurons attenuates fear-induced suppression of feeding. **a** Schematic of microinjection into the PSTN of the C57BL/6J mouse. **b** Schematic illustration of fear-induced suppression of feeding. **c** Cumulative number of small food pellets ingested by mice during the feeding test (Saline, *n* = 4; CNO, *n* = 4). The number of pellets was measured before and after each tone period. **d** The pellet intake during the 1st tone period (Saline, *n* = 6; CNO, *n* = 6). **e** Total food intake during the feeding test session. Each circle represents results from one mouse (Saline, *n* = 5; CNO, *n* = 6). Data are represented as mean ± SEM. *p < 0.05 (unpaired two-sided *t*-test (**d**, **e**)). See also Supplementary Fig. 5.

Recent papers demonstrated that the PSTN neurons are involved in appetite suppression^33,40^. Therefore, we next examined the necessity of the lPB-PSTN pathway in feeding suppression. We pharmacogenetically inhibited PSTN neurons using an inhibitory DREADD. Mice were bilaterally injected with AAV (AAV-CaMKIIα-hM4Di:mCherry) into the PSTN (Fig. 4a). There was no significant difference in general locomotor activity or baseline food intake between mice administered clozapine N-oxide (CNO) or saline (Supplementary Fig. 5a–f). Therefore, inhibition of PSTN neurons is insufficient to affect baseline food intake. These findings are consistent with a recent report^33^.

We hypothesized that the lPB-PSTN pathway would suppress feeding in an aversive environment. Indeed, a recent study demonstrated that the PB neurons are activated during fear-memory retrieval and reduce food intake^2^. We thus took advantage of this recently developed experimental paradigm and investigated whether PSTN functions in fear-induced suppression of feeding. Mice were subjected to fear conditioning training with five pairings of a tone that co-terminated with an electrical foot shock. One day after the fear conditioning, a feeding test was performed in the presence of the conditioned tone. Administration of CNO increased the food intake even in the presence of the conditioned tone (Fig. 4c, d), and the total food intake was also significantly higher in the CNO group compared with that in the control group (Fig. 4e). These results suggest the necessity of PSTN neurons in fear-induced suppression of feeding.

### Inhibition of the lPB-PSTN pathway attenuates fear-induced suppression of feeding

**Fig. 5:**
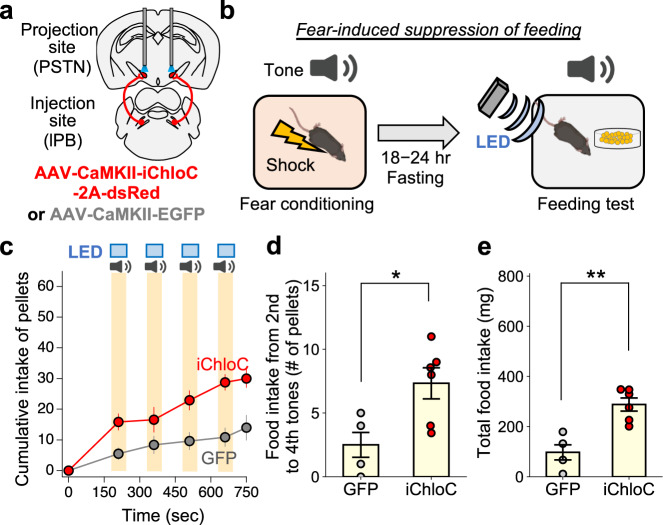
Inhibition of the lPB-PSTN pathway attenuates fear-induced suppression of feeding. **a** Schematic of microinjection and placement of the LED cannula unit of the C57BL/6J mouse. **b** Schematic illustration of fear-induced suppression of feeding. **c** Cumulative number of small food pellets ingested by mice during the feeding test (GFP, *n* = 4; iChloC, *n* = 6). **d** The averaged pellet intake from 2nd tone to 4th tone (GFP, *n* = 4; iChloC, *n* = 6). **e** Total food intake during the feeding test session. Each circle represents results from one mouse (GFP, *n* = 4; iChloC, *n* = 6). Data are represented as mean ± SEM. *p < 0.05; **p < 0.01 (unpaired two-sided *t*-test (**d**, **e**)).

We next examined the necessity of the lPB-PSTN pathway in feeding suppression by optogenetically inhibiting this pathway using the inhibitory opsin iChloC^41,42^. Mice were bilaterally injected with AAV (AAV-CaMKII-iChloC-2A-dsRed or AAV-CaMKII-EGFP) into the lPB (Fig. 5a) and a fear-induced suppression of feeding paradigm was conducted (Fig. 5b). Photostimulation (continuous light for 60 s) was applied to the lPB-PSTN pathway during the tone period. Photostimulations increased the food intake, especially in the period from the second tone to fourth tones (Fig. 5c, d). Total food intake was also significantly higher in the iChloC group (Fig. 5e). These results indicate the necessity of the lPB-PSTN pathway in the suppression of feeding in an aversive environment.

### PACAP-expressing neurons are enriched in the PSTN

**Fig. 6:**
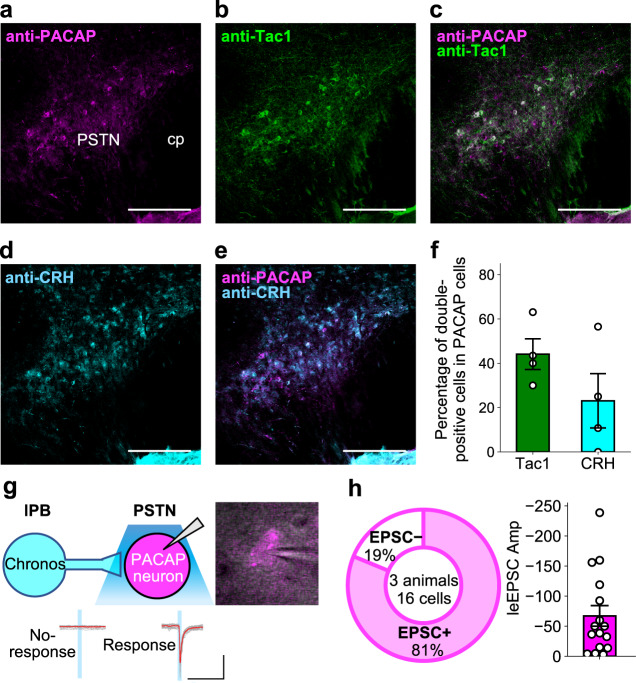
PACAP^PSTN^ neurons overlap both Tac1^PSTN^ and CRH^PSTN^ neurons. **a**–**e** PACAP, Tac1, and CRH immune-positive neurons in the PSTN. Scale bars represent 200 μm. cp, cerebral peduncle. Experiments were repeated independently in at least four mice with similar results. **f** Quantification of the percentage of PACAP neurons co-expressing Tac1 or CRH (*n* = 4 images). It was calculated as the ratio of the number of cells (double-positive cells/PACAP-positive cells). **g** Schematic of the electrophysiological analysis and representative traces of EPSCs (gray, 15 consecutive responses; red, average) evoked by photo-stimulation (every 20 s, 5-ms duration). Scale bar, 100 pA and 50 ms. **h** Proportion of the recorded PACAP^PSTN^ neurons with EPSCs (EPSC–, *n* = 3 cells; EPSC+, *n* = 13 cells) and summary of light-evoked EPSC amplitudes (*n* = 16 cells). Data are represented as mean ± SEM.

To further examine the postsynaptic properties and downstream targets of the lPB-PSTN pathway, we focused on PACAP, which is encoded by the *Adcyap1* gene. PACAP is well characterized in aversive behaviors and feeding behaviors^28,30,43^. Consistent with a previous report^44^, we found that PACAP is highly enriched in the PSTN (Fig. 6a). A recent paper demonstrated two discrete PSTN subpopulations, those that express tachykinin-1 (Tac1) and those that express corticotropin-releasing hormone (CRH). We thus investigated co-expression of PACAP, Tac1, and CRH using immunohistochemical analysis. Some PACAP-expressing neurons (PACAP^PSTN^ neurons) overlapped with Tac1- and CRH-expressing neurons (Fig. 6b–e). We found that 44% of PACAP^PSTN^ neurons expressed Tac1 and 23% of PACAP^PSTN^ neurons expressed CRH (Fig. 6f).

In addition, to identify downstream projections of PACAP^PSTN^ neurons, mice were unilaterally injected with AAV-Syn-FLEX-Chronos:GFP into the PSTN (Supplementary Fig. 6a). We identified at least five projection targets of PACAP^PSTN^ neurons, such as the BNST, CeA, medial reticular nucleus (MRN), PB, and nucleus of the solitary tract (NTS) (Supplementary Fig. 6b–f). These projection targets are overlapped with those of Tac1^PSTN33^.

We further confirmed that the PACAP^PSTN^ neurons indeed received synaptic inputs from the lPB neurons, using the whole-cell patch-clamp recording techniques. Following injection of AAV-Syn-Chronos:GFP and AAV-EF1a-FLEX-hM4Di-2A-cgfTagRFP into the lPBs and PSTNs of *Pacap-IRES-Cre* mice, respectively, light-evoked EPSCs were recorded from over 80% of PACAP^PSTN^ neurons (Fig. 6g, h). These results indicate that a large proportion of the PACAP^PSTN^ neurons received synaptic inputs from the lPB neurons.

### Activation of the PACAP-expressing PSTN neurons promotes real-time avoidance behavior as well as memory formation

**Fig. 7:**
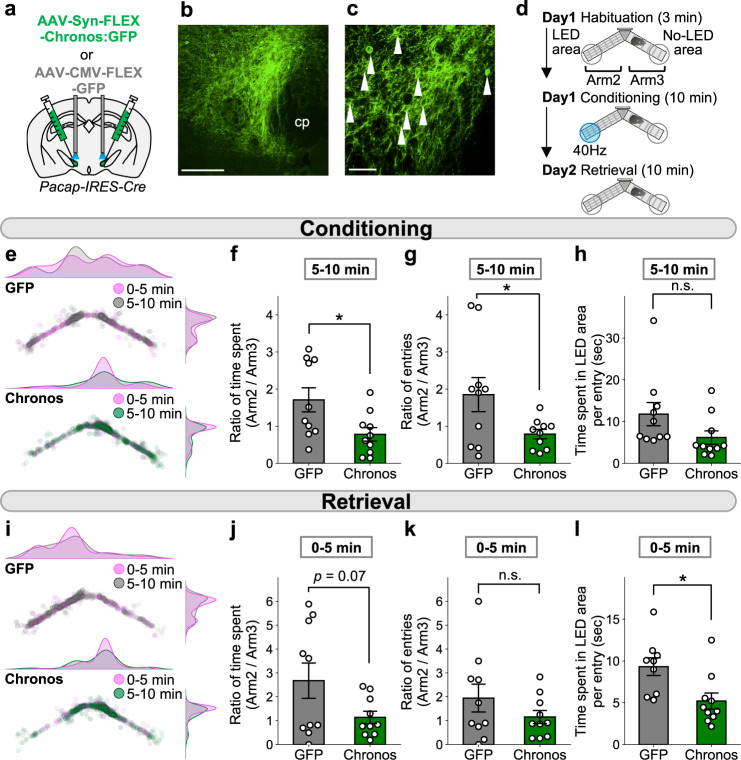
Photoactivation of the PACAP^PSTN^ neurons promotes avoidance behaviors and aversive learning. **a** Schematic illustration of bilateral injection into *Pacap-IRES-Cre* mice. **b**, **c** Representative images of fluorescent at the injection site. Arrowheads indicate the cell bodies of Chronos:GFP-expressing cells. Scale bars represent 200  μm (**b**) or 50 μm (**c**). cp, cerebral peduncle. More than three independent experiments were conducted and similar results were obtained. **d** Experimental schedule of the place avoidance test. **e** Density and scatter plots showing the position of a representative mouse during the conditioning session (top, GFP; bottom, Chronos). **f**–**h** Summary of ratios of time spent in each arm, entries into each arm, and time spent in the LED area per entry during the latter half (5–10 min) of the conditioning session (GFP, *n* = 10; Chronos, *n* = 10). **i** Density and scatter plots showing position of a representative mouse during the retrieval session (top, GFP; bottom, Chronos). **j**–**l** Summary of ratios of time spent in each arm, entries into each arm, and time spent in the LED area per entry during the first half (0–5 min) of the retrieval session (**j**, **k** GFP, *n* = 10; Chronos, *n* = 10, **l** GFP, *n* = 9; Chronos, *n* = 10). Each circle represents results from one mouse. Data are represented as mean ± SEM. n.s., *p* > 0.05; *p < 0.05 (unpaired two-sided *t*-test). See also Supplementary Fig. 7.

To evaluate the physiological relevance of PACAP^PSTN^ neurons at the behavioral levels, AAV-Syn-FLEX-Chronos:GFP or AAV-CMV-FLEX-GFP was injected into the bilateral PSTNs of *Pacap-IRES-Cre* mice, and PACAP^PSTN^ neurons were photostimulated (Fig. 7a–c). First, we performed a real-time place avoidance test using the same Y-maze system as in Fig. 2 (Fig. 7d). Mice were habituated to the apparatus, and they exhibited no bias between the two arms (Supplementary Fig. 7a–c).

In the conditioning session, the ratios of time spent and entries into each arm (i.e., Arm 2/Arm 3) during the latter half (5–10 min) of the observation period were significantly shorter in Chronos mice compared with control mice (Fig. 7e–g). Time spent in the LED area per entry tended to be decreased in Chronos mice (Fig. 7h). These results indicate that activation of the PACAP^PSTN^ neurons promotes avoidance behavior.

The day after the conditioning session, a memory retrieval test in which mice were placed in the same apparatus used for the conditioning session was performed. In addition, the ratios of time spent and entries into each arm (i.e., Arm 2/Arm 3) during the first half (0–5 min) of the observation period tended to be decreased in Chronos mice (Fig. 7i–k). Time spent in the LED area per entry was significantly reduced in Chronos mice (Fig. 7l). Collectively, these results suggest that a memory was formed by activation of the PACAP^PSTN^ neurons.

### Activation of the PACAP^PSTN^ neurons suppresses feeding behavior

**Fig. 8:**
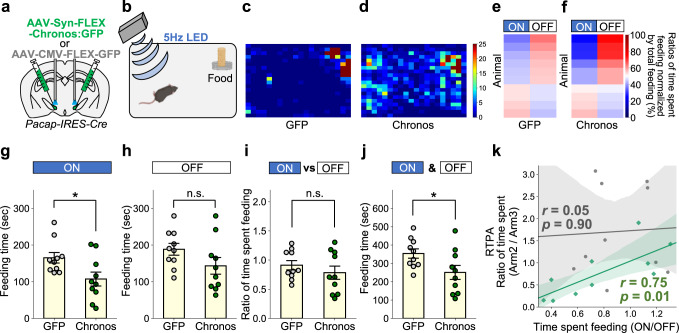
Feeding behavior is suppressed by activation of the PACAP^PSTN^ neurons. **a** Bilateral injection into *Pacap-IRES-Cre* mice. **b** Schematic illustration of the feeding test. **c**, **d** Each heatmap represents the time spent in each area for a representative mouse during the 12-min observation period. **e**, **f** Heatmaps represent ratios of time spent feeding during LED-on (6 min) and LED-off (6 min) to total feeding time for each individual mouse. **g** Time spent feeding during a 6-min LED-on period. **h** Time spent feeding during 6-min LED-off period. **i** Ratio of time spent feeding during LED-on/LED-off periods. **j** Total time spent feeding during the 12-min observation period. Each circle represents results from one mouse (GFP, *n* = 10; Chronos, *n* = 10). Data are represented as mean ± SEM. n.s., *p* > 0.05; *p < 0.05 (Unpaired two-sided *t*-test). **k** Correlations of ratio of time spent feeding during LED-on/LED-off periods and the ratio of time spent in each arm in the real-time place avoidance (RTPA) test in Fig. 5, which would represent feeding and avoidance behavior, respectively. Each dot represents a value obtained for one mouse (gray, GFP; green, Chronos). Regression lines (gray, GFP; green, Chronos) were drawn with 95% confidence intervals. Pearson’s correlation coefficients were shown (YFP, *r* = 0.05, *p* = 0.90; Chronos, *r* = 0.75, *p* = 0.01).

After the real-time avoidance test, we investigated feeding behaviors using the same *Pacap-IRES-Cre* mice (Fig. 8a, b) and the feeding test protocol described above (Fig. 3). Each heatmap represents the time a representative mouse spent in a test chamber during the 12-min observation period with photostimulation (Fig. 8c, d). Ratios of time spent feeding during LED-off (6 min) and LED-on (6 min) to the total feeding time were calculated for individual mice (color-coded in Fig. 8e, f). Moreover, Chronos mice exhibited a significantly lower ratio of time spent feeding during LED-on (6 min) to total feeding time (Fig. 8g), and slightly lower ratio for LED-off (6 min) to total feeding time (Fig. 8h). These results demonstrated that photostimulation of PACAP^PSTN^ neurons elicited a substantial effect on feeding suppression. Intriguingly, the total feeding time was decreased in Chronos mice while the LED-on/LED-off ratio was unchanged (Fig. 8i, j), in contrast to the effect of the lPB-PSTN terminal (i.e., presynaptic) stimulation (Fig. 3j). These results suggest that both pre-synaptic stimulation and post-synaptic stimulation suppressed feeding, but the latter may elicit a longer-lasting effect than the former. We next analyzed the relationship between real-time place aversion test and feeding test results in individual mice. Specifically, we compared the ratio of time spent in each arm (Arm 2/Arm 3) in the place aversion test with the ratio of time spent feeding (LED-on/LED-off) in the feeding test (Fig. 8k), and identified a positive correlation only in Chronos mice (Pearson correlation coefficient: GFP, *r* = 0.05, *p* = 0.90; Chronos, *r* = 0.75, *p* = 0.01).

### Inhibition of PACAP^PSTN^ neurons attenuates aversive memory formation

**Fig. 9:**
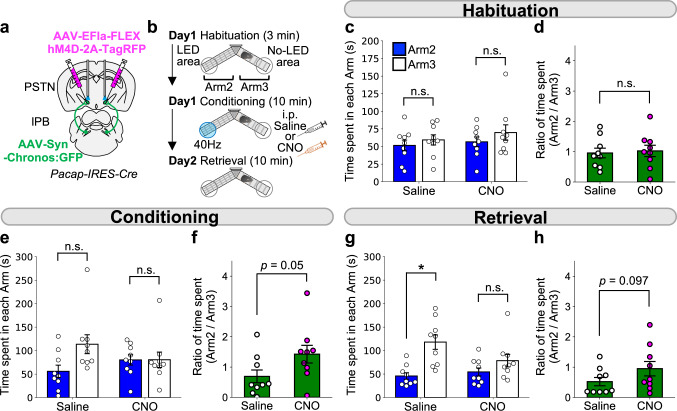
Inhibition of PACAP^PSTN^ attenuated effects of the lPB-PSTN pathway. **a** Schematic of microinjection and placement of the LED cannula unit. **b** Experimental schedule of the real-time place aversion test using C57BL/6J mice. Saline or CNO was injected 30 min before the conditioning session. **c**–**h** Summary of time spent in each arm and summary of ratio of time spent in each arm during the habituation session (**c**, **d**), the conditioning session (5–10 min) (**e**, **f**), and the retrieval session (0–4 min) (**g**, **h**). Each circle represents results from one mouse (*n* = 9). Data are represented as mean ± SEM. n.s., *p* > 0.05; *p < 0.05 (two-sided Wilcoxon matched-pairs signed rank test (**d**, **f**, **h**) and Wilcoxon matched-pairs signed rank test followed by correction with Holm method (**c**, **e**, **g**)).

To evaluate the function of PACAP^PSTN^ neurons as the major targets of lPB-PSTN projections, AAV-Syn-Chronos:GFP and AAV-EFla-FLEX-hM4Di-2A-TagRFP were injected into the bilateral lPBs and PSTNs of *Pacap-IRES-Cre* mice, and axonal terminals of lPB neurons were photostimulated (Fig. 9a). First, we performed a real-time place avoidance test using the same Y-maze system shown in Fig. 2 (Fig. 9b). Mice were habituated to the apparatus and exhibited no bias between time spent in the two arms (Fig. 9c, d). Administration of CNO (1 mg/kg) tended to decrease the bias in time spent between Arm 2 (containing the LED area) and Arm 3 (Fig. 9e, f). These results suggest that inhibition of PACAP^PSTN^ neurons attenuated the acute avoidance behavior induced by lPB-PSTN activation. The day after the conditioning session, a memory retrieval session was conducted. Administration of CNO attenuated the aversive memory compared with mice in the saline group (Fig. 9g, h), suggesting that PACAP^PSTN^ activation is critical for lPB-PSTN-mediated aversive memory formation.

### PACAP signaling is involved in aversive memory formation

**Fig. 10:**
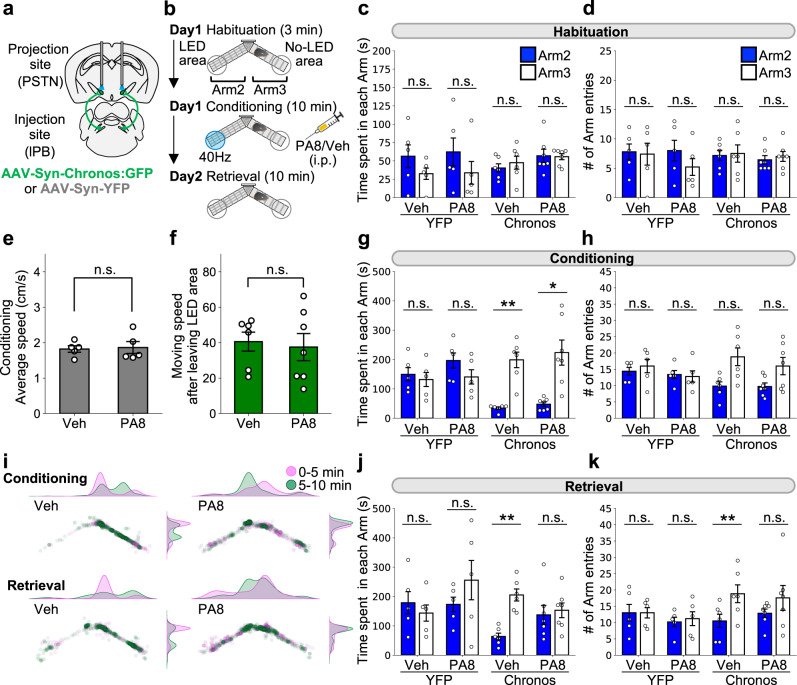
Blockade of PACAP receptor impairs aversive memory induced by the lPB-PSTN. **a** Schematic of microinjection and placement of the LED cannula unit. **b** Experimental schedule of the real-time place aversion test using C57BL/6J mice. The PACAP receptor antagonist PA8 was injected 30 min before the conditioning session. **c**, **d** Summary of time spent in each arm (**c**) and the number of entries into each arm (**d**) during the habituation session. **e** Effects of PA8 administration on averaged locomotion speed of YFP mice during the conditioning session (Veh, *n* = 5; PA8, *n* = 5). **f** Effects of PA8 administration on the escape behavior of Chronos mice during the conditioning session (Veh, *n* = 6; PA8, *n* = 7). **g**, **h** Summary of time spent in each arm (**g**) and the number of entries into each arm (**h**) during 0–10 min of the conditioning session. **i** Density plots and scatter plots showing the position of a representative mouse in a conditioning session (top) and retrieval session (bottom) in Chronos mice. Plots shown are for the same mouse across sessions. **j**, **k** Summary of time spent in each arm (**j**) and the number of entries into each arm (**k**) during 0–10 min of the retrieval session. Each circle represents results from one mouse (YFP-Veh, *n* = 5; YFP-PA8, *n* = 5; Chronos-Veh, *n* = 6; Chronos-PA8, *n* = 7). Data are represented as mean ± SEM. n.s., *p* > 0.05; *p < 0.05; **p < 0.01 (Unpaired two-sided *t*-test (e, **f**) and paired two-sided *t*-test followed by correction with Holm method (**c**, **d**, **g**, **h**, **j**, **k**)). See also Supplementary Fig. 8.

We found that PACAP^PSTN^ neurons induce avoidance behavior and play a key role in aversive memory formation (Fig. 7). We hypothesized that PACAP signaling is involved in the adaptive behaviors induced by the activation of the lPB-PSTN pathway. To test this hypothesis, we applied a pharmacological approach employing the PACAP receptor (PAC1) antagonist, PA8^45,46^. First, we bilaterally injected AAV-Syn-Chronos:GFP or AAV-Syn-YFP into the lPB of C57BL/6 J mice, and implanted dual optic cannulae over the PSTN. Next, the Y-maze aversion test was conducted in the same manner described above (Fig. 2c, d). Mice were habituated to the apparatus, and they exhibited no bias between the two arms (Fig. 10c, d). Then, 30 min before the conditioning session, mice were intraperitoneally injected with either PA8 (30 mg/kg) or vehicle (10% DMSO in saline). PA8 administration did not affect average locomotion speed (Fig. 10e) or escape behavior as indicated by moving speed from the LED (Fig. 10f). Chronos mice exhibited decreased time spent in each arm and the ratio of the number of entries in Arm 2 (containing the LED area) and increased time spent in Arm 3 both in the PA8-administered group and the vehicle control groups (Fig. 10g–i). These results indicate that the acute aversive behavior induced by lPB-PSTN activation was not influenced by blockade of PACAP signaling.

The day after the conditioning session, a memory retrieval session was conducted. Interestingly, the PA8 Chronos mice exhibited no bias for time spent in each arm or the ratio of the number of entries between the two arms, while the vehicle-administered Chronos mice exhibited strong avoidance of Arm 2, suggesting aversive memory retrieval (Fig. 10i–k). These results suggest that PACAP signaling is critical for lPB-PSTN-mediated aversive memory formation.

Using the same mice, a feeding test was conducted following PA8 administration (Supplementary Fig. 8a). No obvious effects on total feeding time or ratios of time spent feeding (LED-on/Total or LED-on/LED-off) were observed (Supplementary Fig. 8b–d). Collectively, our findings suggest that PACAP signaling plays a crucial role in aversive memory formation rather than acute aversive behavior.

We next examined the physiological roles of PACAP signaling in the lPB-PSTN pathway. PACAP38 (10 nM) application did not significantly affect the light-evoked EPSC amplitude (before, average –64 ± 17 pA; PACAP38, average –56 ± 14 pA, *n* = 10 cells; Supplementary Fig. 9a–c) or paired-pulse ratio (PPR) (before, average 0.93 ± 0.09; PACAP38, average 0.74 ± 0.04 pA, *n* = 10 cells; Supplementary Fig. 9d). Application of PACAP38 tended to decrease the number of action potentials induced by current injections (Supplementary Fig. 9e, f), but no detectable changes were observed in the membrane potentials before and after the PACAP38 application (before, averaged –66 ± 2 mV; PACAP38, averaged –66 ± 3 mV, *n* = 9 cells; Supplementary Fig. 9g). These results suggest that PACAP signaling does not directly affect the lPB-PSTN synaptic transmission or membrane excitability of the PSTN neurons. Thus, the most plausible action site of the PACAP would be downstream of the PSTN.

Finally, we investigated the effects of PACAP expressed in the PSTN on aversive behavior induced by activation of the lPB-PSTN pathway using siRNA-mediated knockdown. Chronos-expressing mice were bilaterally injected with PACAP-siRNA or scrambled control siRNA solution into the PSTN and an optic cannulae was implanted over the PSTN (Supplementary Fig. 10a). The Y-maze aversion test was conducted described above (Fig. 2c, d). Neither ratios of time spent nor entries into each area (i.e., LED area/No-LED area) during a conditioning session were different between the control siRNA and PACAP-siRNA groups (Supplementary Fig. 10b, c). Notably, the ratio of time spent was significantly increased and the ratio of entries also tended increase following PACAP-siRNA injection in a memory retrieval session (Supplementary Fig. 10d, e). These results are consistent with those of pharmachological blockade of PACAP signaling (Fig. 10), and support the idea that PACAP released from the PSTN functions in aversive memory formation.

## Discussion

The present study reveals that lPB projections to the PSTN pathway promote avoidance behaviors, aversive learning, and suppress feeding. While neural circuits for feeding have been extensively studied in the hypothalamus, those for controlling suppression of feeding induced by fear and pain are still largely unknown. The lPB receives threat-related signals and transmits them to many brain regions, including the PSTN in the lateral hypothalamus. The present study sheds light on neuronal mechanisms underlying fear-induced anorexia.

A growing body of evidence suggests that the lPB in the pons serves as the alarm center^7,8^ for transmitting noxious sensory information to multiple downstream targets, such as the PSTN, VPM, VMH, CeA, and BNST^19,36,37,47^. We confirmed the projection patterns of the lPB neurons in the present study using a whole-brain imaging system (Fig. 1c, d and Supplementary Fig. 1; Supplementary Movie 1). Our findings are consistent with a recent report showing that activating CGRP-expressing lPB terminals in the PSTN induces real-time aversion and pseudo fear conditioning^36^. Notably, our results exhibit real-time escaping (Fig. 2i–l), while theirs show real-time freezing. This apparent contradiction is most likely attributable to the selective stimulation of GCRP-positive neurons and/or use of a heterozygous CGRP knockout Cre-mouse line in the previous study. Investigating whether these behavioral differences are attributable to the qualitative (i.e., cell-types) or quantitative (cell-numbers) differences is of future interest.

The PSTN is a relatively unexplored brain region, yet has recently emerged as a critical interface between interoception and emotions^32^. Indeed, a recent study by Carter’s group demonstrated that inhibition of the PSTN neurons expressing tachykinin-1 attenuates the anorexigenic effects of appetite-suppressing hormones with no effect on the baseline food intake^33^. Furthermore, de Araujo’s group recently demonstrated that PSTN is activated by glucagon-like peptide-1 and that optogenetic activation of the PSTN to pontine parvocellular reticular formation pathway inhibits food intake^48^. These findings are consistent with our present study, and these lines of evidence support the notion that the PSTN is involved in the interplay between appetitive and defensive behaviors.

The suppression of feeding could be attributable to a hedonic effect, i.e., mice may have stopped eating because of gratification. Indeed, the PSTN is activated by refeeding after fasting^39,49^. However, this is not likely because lPB-PSTN activation decreased the number of lever-press behaviors (Supplementary Fig. 3). The lPB-PSTN and PSTN are involved in stress-related feeding suppression (Figs. 4, 5). These results suggest that lPB-PSTN activation did not serve as a hedonic signal (e.g., palatability) for the satiety network, but rather served as an aversive signal (e.g., malaise) for the satiety network to suppress feeding.

We performed optogenetic activation of the lPB-PSTN pathway. Because it is known that lPB neurons send collateral projections to various targets, we cannot completely rule out the possibility of antidromic activation. However, recent studies suggest that terminal stimulation does not produce robust antidromic activation^36,37^. We also demonstrated that the effects of lPB-PSTN pathway activation were attenuated by inhibition of PACAP^PSTN^. Therefore, the behavioral effects observed in the present study are more likely due to pathway-specific manipulation rather than nonspecific collateral effects.

Abundant PACAP-expressing neurons were detected in PSTN (Fig. 6a) and received input from lPB neurons (Fig. 6g, h). We investigated the effects of chemogenetically inhibiting PACAP^PSTN^ neurons during stimulation of the lPB-PSTN pathway and found that both the avoidance behavior and aversive memory formation induced by activation of the lPB-PSTN pathway were attenuated (Fig. 9). These results suggest that the aversive signal from lPB is transmitted via PACAP^PSTN^ to induces aversive behaviors and suppression of feeding.

PACAP-expressing neurons are also enriched in other hypothalamic regions, such as the PVH and VMH. PACAP-expressing neurons in the PVH promote feeding behaviors, while those in the VMH suppress feeding behaviors^50,51^. PACAP^PSTN^ and Tac1^PSTN^ neurons both suppress feeding behavior (Fig. 8)^33^. Therefore, to regulate of feeding behavior, PACAP neurons in the hypothalamus may serve different roles in different brain regions.

To examine the downstream circuit of PSTN, we observed projection targets of PACAP^PSTN^ neurons and identified at least five target regions: BNST, CeA, MRN, PB, and NTS. Some of these target regions have reportedly critical roles in avoidance behavior and feeding behaviors^18,52^. Recent studies demonstrated that Tac1^PSTN^ neurons also project to these target regions and suppress feeding behavior^33^. Thus, PACAP^PSTN^ is involved in transmitting the aversive signal to downstream targets and regulates avoidance behavior and feeding behaviors. In addition, it is possible that the projection from the lPB to PSTN can provide some form of feedback on itself. Feedback loops and plasticity mechanisms downstream of the PSTN are essential topics for future investigation.

PACAP is highly enriched in the PSTN (Fig. 6a)^53^, and activation of PACAP-expressing PSTN neurons induced strong real-time aversion, fear memory formation, and suppression of feeding (Figs. 7 and 8). Notably, markers enriched in the PSTN, such as PACAP, tachykinin-1, and corticotropin-releasing factor, are also postulated to be involved in stress-related affective and motivational behaviors^22,33,54,55^. To explore molecular mechanisms underlying the regulation of circuits by PACAP, we systemically applied the PACAP receptor antagonist PA8 before lPB-PSTN photo-stimulation experiments. PA8 administration almost completely blocked fear memory formation, but had no detectable effect on real-time aversion (Fig. 10g–k). The ability of PACAP to enhance synaptic plasticity and improve memory and learning has been characterized in many brain regions, such as hippocampus and amygdala^56,57^. Therefore, we hypothesized that aversive memory formation induced by lPB-PSTN pathways may depend on the PACAP signaling-mediated plasticity. Neither lPB-PSTN EPSC amplitude nor PSTN neuronal excitability was affected by bath application of PACAP38 (Supplementary Fig. 9), so they are unlikely to be the PACAP targets. We found that knockdown of PACAP in the PSTN using siRNA attenuated aversive memory formation (Supplementary Fig. 10). These results suggest that PACAP released from PSTN neurons is involved in aversive memory formation. The PACAP receptor, PAC1, is widely expressed in the brain, including PSTN, as well as other PSTN targets such as the CeA and PB [Allen Mouse Brain Atlas^58^]. PACAP is also involved in the synaptic plasticity in the CeA^56^. Collectively, PACAP released from the PSTN may modulate the synaptic plasticity of targets downstream of PSTN, and regulate aversive memory formation. Thus, it is of future interest to dissect out target pathways and molecular mechanisms of PACAP for the regulation of aversive memories.

Our study suggests that the lPB-PSTN network plays a key role in the interplay between feeding and aversion. These findings advance the current understanding of how stress and fear affect motivation and learning through actions on PACAP neurons in the PSTN. Further discernment of precise circuit and molecular mechanisms is crucial for future discovery and development of new therapies for eating disorders, such as anorexia and bulimia, which are considered to arise from dysregulation of feeding behaviors independent of homeostatic nutritional states.

## Methods

### Animals

All the experimental protocols in this study including the use of animals were approved by the Institutional Animal Care and Use Committee of the Jikei University (Tokyo, Japan; Approval No. 2018-030, 2019-010). All experiments complied with the Guidelines for Proper Conduct of Animal Experiments by the Science Council of Japan (2006) and those recommended by the International Association for the Study of Pain. All efforts were made to reduce the number of animals used and the suffering of the animals. Male C57BL/6J mice (Japan SLC, Shizuoka, Japan) and male and female *Pacap-IRES-Cre* mice (generous gift from Prof. Bradford B. Lowell, Harverd University)^50^ were group-housed in temperature (20–24 °C) and humidity (45–65%) controlled environments on a 12 h light/dark cycle and provided with food and water ad libitum. *Pacap-IRES-Cre* mice were derived from fertilized eggs (generously provided by Dr. Ken-ichiro Nakajima, National Institute for Physiological Sciences) by CLEA Japan and backcrossed for at least four times to C57BL/6J.

### Adeno-associated virus (AAV)

C57BL/6J mice were used for viral injections with AAV1-hSyn-Chronos:GFP (purchased from UNC Vector Core), AAV5-hSyn-eGFP (purchased from University of Pennsylvania Vector Core), AAVDJ-hSyn-EYFP (a generous gift from Prof. Toshihisa Ohtsuka, University of Yamanashi), AAVDJ-CaMKIIα-hM4Di:mCherry, AAVDJ-CaMKIIα-EGFP, and AAVDJ-CaMKIIα-iChloC-2A-dsRed. *Pacap-IRES-Cre* mice were used for viral injections with AAV1-hSyn-FLEX-Chronos:GFP (purchased from UNC Vector Core), AAV2/1-CMV-FLEX-GFP (a generous gift from Prof. Hiroyuki Hioki, Juntendo University), or AAVDJ-EF1a-FLEX-hM4Di-2A-cgfTagRFP (a generous gift from Prof. Kazuto Kobayashi, Fukushima Medical University).

### Stereotaxic surgery

Four- or five-week-old male C57BL/6J and five- or six-week-old *Pacap-IRES-Cre* mice were intraperitoneally anesthetized with a mixture of medetomidine hydrochloride (0.75 mg/kg; Zenoaq, Fukushima, Japan), midazolam (4.0 mg/kg; Astellas, Tokyo, Japan), and butorphanol tartrate (5.0 mg/kg; Meiji Seika Pharma, Tokyo, Japan) and fixed in a stereotaxic device. Microinjections into the PB were carefully targeted to the lateral PB (lPB). The AAV (0.25 μl) was microinjected bilaterally into the lPB (6.4 mm posterior to bregma, 1.25 mm lateral to midline, and 3.2 mm ventral to the skull surface, with a 20° anterior-to-posterior angle to avoid damaging the superficial arteries during surgery) using a Hamilton microsyringe (1701RN Neuros Syringe, 33 G, 10 μl; Hamilton Company, Reno, NV, USA)^10^. The AAV (0.30 μl) was microinjected bilaterally into the PSTN (1.7 mm posterior to bregma, 1.1 mm lateral to midline, and 4.9 mm ventral to the skull surface), The injection speed (50 nl/min) was controlled by a microsyringe pump (UMP3; UltraMicroPumpII with SYS-Micro4 Controller, UMP2, UMC4; World Precision Instruments, Sarasota, FL, USA). Injection syringes were left in place for 10 min before withdrawing. Expression in lPB was confirmed for all animals used in this study.

After 3–5 weeks, a second surgical procedure was performed for the placement of a bilateral light-emitting diode (LED) cannula unit consisting of dual optical fibers (0.25 mm in diameter, 4.5 or 4.8 mm in length, and 2.2 mm in spacing) attached to a LED body (blue, 470 nm) (TeleLCD-B-4-250-5; Bio Research Center, Tokyo, Japan). The LED cannula unit was stereotactically inserted to target the PSTN (1.8 mm posterior to bregma), and fixed to the skull with dental cement (GC Fuji I; GC Corporation, Tokyo, Japan). Mice were allowed to recover for several days.

### Whole-brain imaging using FAST

Male C57BL/6J mice were deeply anesthetized with an intraperitoneal injection of a mixture of midazolam (4 mg/kg), medetomidine (0.75 mg/kg), and butorphanol tartrate (5 mg/kg) and perfused transcardially with 4% paraformaldehyde. Brains fixed with 4% paraformaldehyde (Nacalai Tesque, Kyoto, Japan) were embedded in 4% agarose gel (Nacalai Tesque) dissolved in phosphate-buffered saline (PBS). Whole-brain fluorescence images were acquired with a block-face serial microscopy tomography (FAST) system^34,35^. The FAST system is composed of 488 nm laser (OBIS 488 LS; Coherent, Santa Clara, CA), a sCMOS camera (Zyla4.2 Plus; Andor Technology, Belfast, UK), a Nipkow disk-based confocal scanner unit (CSU-W1; Yokogawa Electric Co., Tokyo, Japan), piezo positioning system for objective positioning (P-725.2CD PIFOC; Physik Instrumente, Karlsruhe, Germany), 16× water immersion objective lens (CFI75 LWD 16×W; Nikon Instruments, Tokyo, Japan), upright microscope frame (ECLIPSE FN1; Nikon Instruments), xy stage with linear encoder (TI-S-ER; Nikon Instruments), microslicer (LinierSlicer, MH-1; Dosaka EM, Tokyo, Japan), high-accuracy linear actuators (PA180S-200X50Z50W100Y-1353; COMS, Amagasaki, Japan), and CP-700 controller (COMS). Optical z-stacks (5 μm z-spacing) were imaged as a mosaic of fields of view with 20% overlap in the x-y plane. The x-y plane section images were then reconstructed from the field-of-view tiles using an in-house all-in-one stitching program, FASTitcher^35^. Z-projection (maximum intensity) was performed to stack 17 sequential images using FASTitcher.

### Y-maze place aversion test

All behavioral training and testing were conducted in a custom-built Y-shaped maze apparatus (YM-3002; O’Hara & Co., Ltd., Tokyo, Japan) placed in a sound-attenuating chamber (CL-M3; O’Hara & Co., Ltd.) (Fig. 2c)^11^. The Y-maze consists of three arms with different textures (Arm 1, punched metal; Arm 2, grid metal; Arm 3, mesh metal). Three to five days before conditioning, adult male C57BL/6J mice were habituated daily to dummy Teleopto-receivers (2 g, TeleDummy; Bio Research Center) attached to their heads and handling for several minutes. In the habituated session, mice were habituated to the Y-maze apparatus (10 lux, 50 dB background white noise), in which a divider was inserted in Arm 1 so that a mouse could only freely move between Arm 2 and Arm 3, and the behavior was monitored for 3 min. More than two hours after the habituation session, the conditioning session was conducted in a similar manner as the habituation session. Teleopto-receiver (2 g, TeleR-2-P, an infrared-driven wireless LED unit; Bio Research Center) was attached to the LED cannula unit immediately before the conditioning session. The LED stimulating program was designed by the Time OFCR1 software (O’Hara & Co., Ltd.) and a programmable stimulator (Master-8; A.M.P. Instruments Ltd., Jerusalem, Israel); specifically, whenever the mouse entered the LED area in Arm 2 (shown as a blue circle in Fig. 2c), it received 5 ms of LED illumination (40 Hz) that was controlled by an infrared-driven remote controller (Teleopto remote controller; Bio Research Center). For comparison, a non-illuminated control area (no-LED area; shown as a gray circle in Fig. 2c) was set on the opposite side in Arm 3 as the exact same size as the LED area. Mouse behavior was monitored for 10 min. The next day, a retrieval session was conducted in the same manner as the conditioning session for 10 min, while no LED illumination was applied.

Mouse behavior was captured using a digital camera at 2 frames per second. Time spent in each arm, LED and no-LED area, and the number of each arm entry was analyzed using Time OFCR1 software. Moving speed for 3 s after leaving the LED area was calculated from the coordinates of the center of gravity. Trials that returned to the LED area within 3 s were excluded.

In chemogenetical inhibition of PACAP^PSTN^ neurons during stimulation of the lPB-PSTN pathway (Fig. 9), the place aversion test was performed for four days. Different floor contexts were used for Day 1–2 (Arm 1, punched metal; Arm 2, grid metal; Arm 3, mesh metal) and Day 3–4 (Arm 1, grid metal; Arm 2, mesh metal; Arm 3, punched metal). Administration of saline or CNO was alternated for each mouse.

### Feeding test

The feeding tests using optogenetics were conducted in a mouse chamber which was the same size and shape as the home cage, with a little amount of bedding and a conventional food pellet (3–5 g) glued on a 35-mm dish which was fixed on a floor (Fig. 3a). The habituation session of the feeding test was started 4–8 days after the completion of the place avoidance test. Before the feeding tests, 15-min habituation session was performed in the test chamber for consecutively two days. After an overnight fasting period of 18–22 h, a feeding test was conducted in which a mouse was allowed to access a food pellet for 15 min in a test chamber.

For optogenetic manipulation, each mouse was attached to the Teleopto receiver immediately before the assay, and received 5 ms of LED illumination (five 5-Hz pulses with a 5-s inter-train-interval) as described above. Behavioral data were recorded and analyzed using TimeFZ software (O’HARA & CO., Ltd., Tokyo, Japan). Feeding behavior was also captured from the side of the chamber using a web camera (HD Webcam C525, Logicool) and its software (version 2.51, Logicool). Feeding behavior was recorded for 15 min, and 12 min of data starting after one minute were analyzed. Feeding behavior was manually measured using images (~2 frames per second) and defined as the time when the tip of the nose of the mouse was in contact with the fixed food.

Baseline food intake after saline or CNO (1 mg/kg) administration was measured in a mouse chamber (TP-107A; TOYO-LABO Co., Ltd.). Habituation session was performed (10 min, 0 lux, a little amount of bedding), followed by a feeding test was conducted in which a mouse was allowed to access a conventional food pellet (3–5 g) for 3 h. The pellet was replaced with fresh one every hour.

### Fear-induced suppression of feeding test

Fear-induced suppression of feeding test was performed^2^. The Fear conditioning training was performed the day before the feeding test. Adult male C57BL/6J mice were placed in a conditioning chamber (170 mm width × 100 mm depth × 100 mm height, 200 lux, 60 dB background white noise). Following to 3-min habituation period, the mice were conditioned with five pairings of a 20 s tone (4 kHz, 65 dB) that co-terminated with 1 s of foot-shock (0.6 mA) with 40 s inter-stimulation interval. Mice were then fasted for 18–24 h.

In the pharmacogenetical manipulation, mice were i.p. injected with either saline or CNO (1 mg/kg) 30 min before the tone-induced suppression of feeding test. Mice were placed in a chamber (TP-107A; TOYO-LABO Co., Ltd.) without bedding and allowed to access a food cup filled with small food pellets (1811213, TestDiet). During the 750 s feeding test, four tones (4 kHz, 65 dB) were delivered for fear memory retrieval, each 60 s, with 90 s inter-stimulation interval. Behavioral data were recorded and analyzed using TimeFZ software (O’HARA & CO., Ltd., Tokyo, Japan). The number of food pellets was manually counted using ImageJ Fiji (version 1.0) software.

In the optogenetical manipulation, Teleopto-receiver was attached to the LED cannula unit immediately before the feeding test. The LED stimulating program was designed by the TimeFZ software and a programmable stimulator (Master-8). Mice were received 60 s of continuous LED illumination at the same time as tone presentation.

### Lever press test

Each adult male C57BL/6J mouse was placed in an operant task chamber (OPR-3002; O’Hara & Co., Ltd.) and trained to press a lever to obtain a small sucrose reward pellet (1811213, TestDiet). Mice were food deprived for 18–24 h before training sessions and they were allowed to access to conventional food for 2 h after training sessions. The training procedure consists of 1-day magazine training and 3-day operant training. In the 15-min magazine training (Day 1), each mouse was randomly presented a food pellet immediately after a tone. On Days 2–4, each mouse were presented a food pellet immediately after a tone when it pressed a lever in the 30-min training session. During Day 4 training, each mouse pressed a lever more than 20 times.

On Day 5, optogenetic manipulation was performed for 20 min. Using operant learning by training up to Day 4, LED-stimulation was presented instead of a reward food pellet. Mice were food-deprived for 18–24 h before the test as in the traning sessions, and attached to the Teleopto receiver immediately before the test. Each mouse received 5 ms of LED illumination (10 pulses at 20 Hz) without the food pellets or the tone when it pressed a lever. The software (Operant Task Studio V2, O’HARA & CO., Ltd., Tokyo, Japan) was used to measure the number and timing of lever presses.

### Acute brain slice preparation

At least five weeks after AAV injection, adult male C57BL/6J mice or *Pacap-IRES-Cre* mice were deeply anesthetized with isoflurane (5% in 100% O_2_) and the block of the brain containing the PSTN was dissected in an ice-cold cutting solution containing (in mM) 2.5 KCl, 0.5 CaCl_2_, 10 MgSO_4_, 1.25 NaH_2_PO_4_, 3 sodium pyruvate, 92 N-Methyl-D-glucamine, 20 HEPES, 12 N-acetyl-L-cysteine, 25 D-glucose, 5 L-ascorbic acid and 30 NaHCO_3_ equilibrated with 95% O_2_ plus 5% CO_2_. Coronal brain slices (300 μm) were cut with a vibratome (VT1200S, Leica) and kept in the same cutting solution at 34 °C for 10–15 min. Slices were transferred to the 20–25 °C standard artificial cerebrospinal fluid (ACSF) containing (in mM): 125 NaCl, 3 KCl, 2 CaCl_2_, 1.3 MgCl_2_, 1.25 NaH_2_PO_4_, 10 D-glucose, 0.4 l-ascorbic acid and 25 NaHCO_3_ (bubbled with 95% O_2_ + 5% CO_2_) until the recording.

### Electrophysiology

Whole-cell patch clamp recordings were obtained from PSTN neurons, which were visually identified under an upright microscope with oblique illumination (BX-51WI, Olympus) as previously reported^59^. Each slice was continuously perfused with standard ACSF at a flow rate of 1.5–2.5 mL/min at 25 °C in a recording chamber. Patch-clamp electrodes (4–8 MΩ) were made of borosilicate glass pipettes (1B150F-4, World Precision Instruments). The internal solution contained (in mM) 122.5 potassium gluconate, 10 HEPES, 17.5 KCl, 0.2 EGTA, 8 NaCl, 2 MgATP, and 0.3 NaGTP (pH 7.2 as adjusted with KOH; osmolarity, ~290–300 mOsm/kg). Light-evoked EPSCs were recorded at a holding potential of –60 mV in the ACSF containing 100 μM picrotoxin. Chronos was activated by LED illumination system (455 or 470 nm; 455L3 or 470L3, Thorlabs) through a 40× water-immersion objective lens (LUMPLFLN40XW, NA 0.8; Olympus). Photo-stimulation was controlled by Master 8 (A.M.P.I.; pulse duration, 5 ms; every 20 s). Paired-pulse ratio was defined as the ratio of the second EPSC amplitude to the first EPSC amplitude, in response to two stimuli with a 100-ms interstimulus interval. Firing patterns were classified into three types: late-spiking showing a noticeable delay of firing after depolarizing current injection (3 steps with +25 pA increment; 1 s); adapting showing complete or high spike frequency adaptation; regular-spiking which the others defined as. The membrane current and membrane potential were recorded with an Integrated Patch Amplifier (Sutter Instrument) or a MultiClamp 700B amplifier (Molecular Devices), filtered at 2 kHz and digitized at 10 kHz with a 16-bit resolution using a PowerLab interface (AD Instruments), together with timing pulses for photo-stimulation. Tetrodotoxin (TTX; 1 μM; abcam), 4-Aminopyridine (4AP; 100 μM; Sigma), kynurenic acid (Kyn; 3 mM; Sigma) and PACAP38 (137061-48-4, Tocris) were added to the ACSF containing picrotoxin (100 μM; Sigma and FUJIFILM Wako Pure Chemical Corporation) and bath applied. PACAP38 was dissolved in DMSO at 50 μM, kept frozen at –30 °C and then dissolved in ACSF at 10 nM. The recorded membrane currents and membrane potentials were analyzed offline with Igor Pro 7 (WaveMetrics, Portland, OR, USA).

### Retrograde labeling

Alexa Fluor 555-conjugated cholera toxin subunit B (CTB555; Thermo Fisher Scientific, Waltham, MA, USA; C34776) were stereotactically injected (0.30 μl, 0.2% in PBS) into the PSTN (AP –2.3 mm, ML ± 1.1 mm, DV 5.1 mm) of male C57BL/6J mice with the same instruments as described above for stereotaxic surgery. After more than 10 days, the retrogradely labeled mice were deeply anesthetized with an intraperitoneal injection of a mixture midazolam (4 mg/kg), medetomidine (0.75 mg/kg) and butorphanol tartrate (5 mg/kg) and perfused transcardially with 4% paraformaldehyde. The sections were mounted on slides with ProLong™ Diamond Antifade Mountant with DAPI (Thermo Fisher, P36966).

### Axonal projection mapping of PACAP^PSTN^ neurons

The projection sites of PACAP^PSTN^ were investigated using *Pacap-IRES-Cre* female mice. The *Pacap-IRES-Cre* mice were unilaterally injected with AAV-Syn-FLEX-Chronos:GFP into the PSTN. Coronal sections (50 μm) were collected and mounted with encapsulated material (P36966, Invitrogen). Fluorescent images of the injection site and projection sites were acquired using a confocal microscope (FV3000, Olympus) with 488 nm excitation laser light. Each brain region was identified using the Allen Brain Reference Atlas (http://atlas.brain-map.org/).

### Immunohistochemistry

For staining of PACAP-expressing neurons, 350 nL colchicine (40 µg/µL in saline) was injected intracerebroventricularly under a mixture midazolam (4 mg/kg), medetomidine (0.75 mg/kg) and butorphanol tartrate (5 mg/kg) anesthesia. After more than two days, the male C57BL/6J mice were deeply anesthetized with an intraperitoneal injection of a mixture midazolam (4 mg/kg), medetomidine (0.75 mg/kg) and butorphanol tartrate (5 mg/kg) and perfused transcardially with 4% paraformaldehyde. After perfusion, coronal section (50 μm) were cut with a vibratome (VT1000, Leica). As primary antibodies, a rabbit polyclonal antibody against PACAP-38 (1:200, BMA, Rheinstrasse, Switzerland; T-4473), a mouse monoclonal antibody against Substance P/Tac1 (1:200, abcam, Cambridge, UK; ab14184), and a guinea pig polyclonal antibody against CRF (1:300, BMA, Rheinstrasse, Switzerland; T-5007) were used. Sections were incubated two overnight at 37 °C with the primary antibodies in 0.05 M Tris-buffered saline (TBS) containing 10% normal goat serum, 2% bovine serum albumin and 0.5% Triton X-100. After washing in PBS, sections were incubated with a goat anti-rabbit biotinylated antibody (1:200, Vector Laboratories, Newark, USA; BA-1000-1.5) in 0.05 M TBS containing 10% normal goat serum, 2% bovine serum albumin and 0.5% Triton X-100 at room temperature for 2 h. Subsequently washing in PBS, sections were incubated with a goat anti mouse AlexaFluor 488, a goat anti gunia pig AlexaFluor 647 (1:200, Thermo Fisher Scientific, MA USA; A-11029, A-21450) and Streptavidin, AlexaFluor 594 (1:500, Thermo Fisher Scientific, MA USA; S11227) in 0.05 M TBS containing 10% normal goat serum, 2% bovine serum albumin and 0.5% Triton X-100 at room temperature for 2 h. Sections were mounted on slides with ProLong™ Glass Antifade Mountant (Thermo fisher, P36980). Fluorescent images of the triple immuno-staining were acquired using a confocal microscope (FV3000, Olympus) with 488/594/647 nm excitation laser light. Quantification of co-expression of the neuropeptides in the PSTN was analyzed using ImageJ Fiji (version 1.0). Percentage of PACAP cells co-expressing each PSTN marker was calculated as the ratio of the number of cells (double positive cells/PACAP-positive cells).

### PAC1 antagonist (PA8) administration

PA8 (RSD-6657, R&D) was dissolved in DMSO at 88 mM, and then adjusted to 8.8 mM PA8 by addition of saline. The real-time place aversion test with PA8 administration was conducted more than three days after the feeding test without PA8 administration. The real-time place aversion test consisted of three sessions (habituation, conditioning, and retrieval) as mentioned above. Mice were injected (i.p.) with either PA8 (30 mg/kg) or vehicle (10% DMSO in saline) 30 min prior to the conditioning session. The habituation session of the feeding test was started 5–8 days after the completion of the place aversion test with PA8 administration. PA8 was administered 30 min prior to the feeding test.

### siRNA injection

Adcyap1 (mouse)−3 unique 27mer siRNAduplexes (SR404157, OriGene Technologies, Inc.) or 27mer scrambled negative control siRNA (SR30004, OriGene Technologies, Inc.) was dissolved in RNase free resuspension buffer (SR30005, OriGene Technologies, Inc.). Each solution was mixed with 10% glucose solution and in vivo jetPEI (101000040, Polyplus-transfection SAS)^60^. The siRNA solution (0.30 μl) was microinjected bilaterally into the PSTN (1.8 mm posterior to bregma, 1.1 mm lateral to midline, and 4.9 mm ventral to the skull surface) of male C57BL/6 J mice. After injection, the placement of a bilateral LED cannula unit (0.25 mm in diameter, 4.5 mm in length, and 2.2 mm in spacing) attached to a LED body (TeleLCD-B-4-250-5) was performed. The LED cannula unit was stereotactically inserted to target the PSTN (1.8 mm posterior to bregma), and fixed to the skull with dental cement (GC Fuji I; GC Corporation, Tokyo, Japan). Mice were allowed to recover for overnight. Y-maze place aversion test was performed a day after injection.

### Statistical analysis

Statistical analyses were performed using GraphPad Prism 9 software (GraphPad Software, La Jolla, CA). Differences were considered statistically significant at *p* < 0.05. Graphs were created using the GraphPad Prism, the Igor, and the Python software. See also Supplementary Table 1.

### Reporting summary

Further information on research design is available in the Nature Portfolio Reporting Summary linked to this article.

## Supplementary information

Supplementary Information

Description of Additional Supplementary Files

Supplementary Movie 1

Supplementary Movie 2

Supplementary Movie 3

Reporting Summary

## Data Availability

The authors declare that all data supporting the findings of this study are available within the article and its Supplementary Information files. The underlying data of all figures are provided in the Source Data file. All detailed outcomes of the statistical test are provided in Supplementary Table 1. Further information and requests for resources and reagents should be directed to and will be fulfilled by the lead contact, Ayako M. Watabe (awatabe@jikei.ac.jp). Source data are provided with this paper.

## Source data

Source Data
