# Supplementary material for: Parabrachial-to-parasubthalamic nucleus pathway mediates fear-induced suppression of feeding in male mice
Source: Nat Commun. 2022 Dec 30;13:7913. doi: 10.1038/s41467-022-35634-2 (PMC9803671; doi:10.1038/s41467-022-35634-2)
Supplement: Supplementary file 1 — Supplementary Information [file 41467_2022_35634_MOESM1_ESM.pdf]

Supplementary Information

Parabrachial-to-parasubthalamic nucleus pathway mediates fear-induced suppression of feeding in male mice

Takashi Nagashima, Suguru Tohyama, Kaori Mikami, Masashi Nagase,
Mieko Morishima, Atsushi Kasai, Hitoshi Hashimoto, and Ayako M. Watabe*

* Corresponding author. E-mail: awatabe@jikei.ac.jp

This PDF file includes:

Supplementary Figure 1-10

Supplementary Table 1

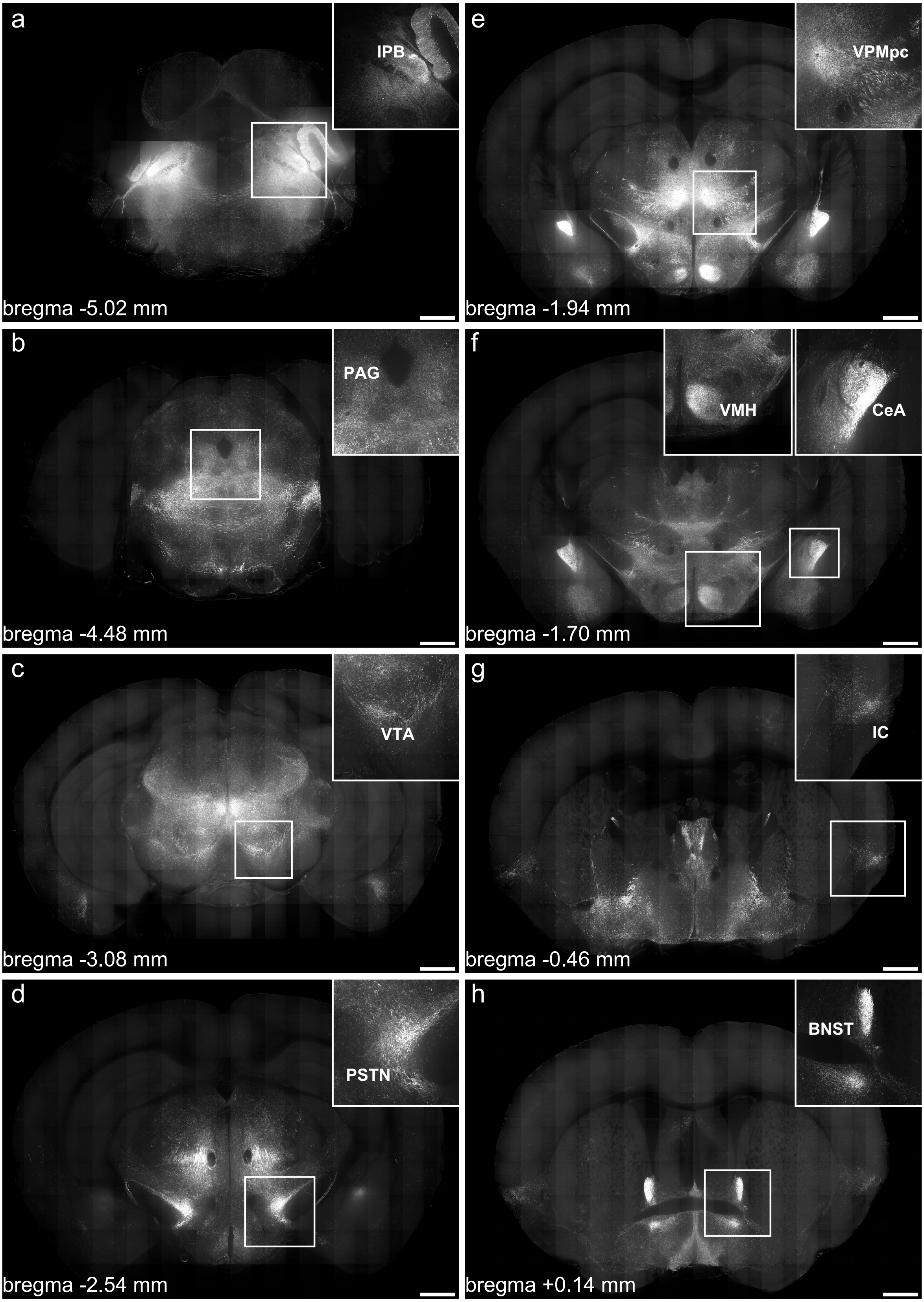

Supplementary Fig. 1 Brain-wide projection of the lPB neurons. a–h Representative images of Chronos:GFP-labeled lPB neurons in different brain regions. Each scale bar represents 1 mm. lateral parabrachial nucleus (lPB), periaqueductal gray (PAG), ventral tegmental area (VTA), parasubthalamic nucleus (PSTN), ventral posteromedial thalamic nucleus (VPMpc), central amygdala (CeA), ventromedial hypothalamus (VMH), insular cortex (IC), and bed nucleus of stria terminalis (BNST). The brightness and contrast were adjusted to show each image optimally. Experiments were repeated independently in at least two mice with similar results.

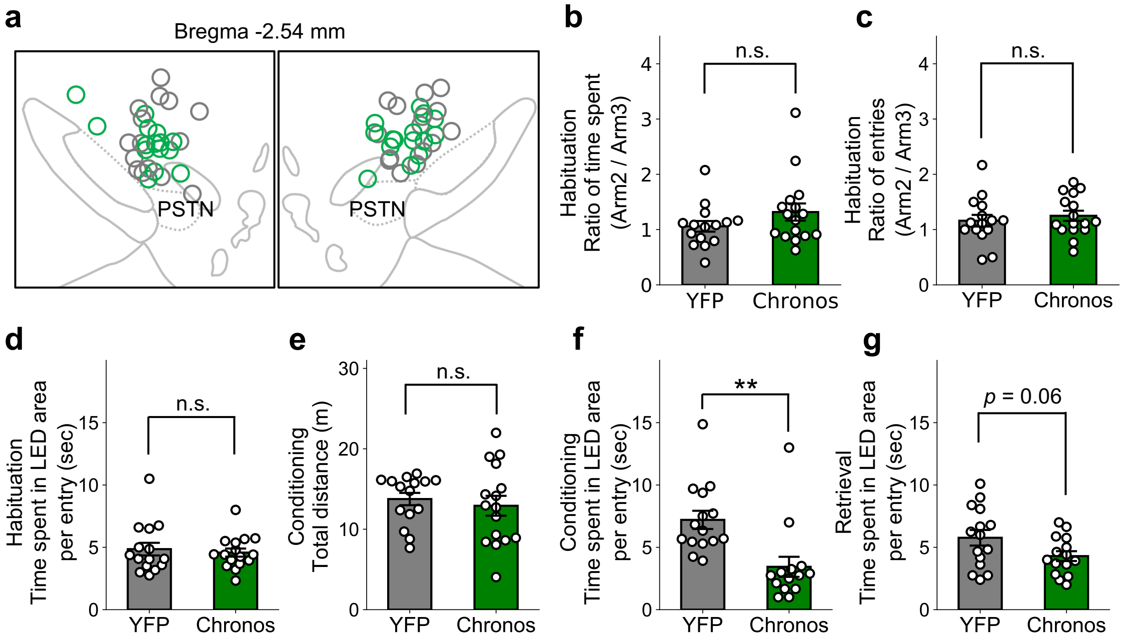

Supplementary Fig. 2 Photoactivation of the lPB-PSTN promotes avoidance behavior. a Schematic illustration of the PSTN and the approximate positions of the LED cannula tips (gray, YFP mice; green, Chronos mice). One specimen in Chronos mice was excluded because of the indistinct tip. **b–d** Summary of ratios of time spent in each arm, entries into each arm, and time spent in the LED area per entry during the habituation session (YFP, n = 15; Chronos, n = 16). **e, f** Total distance (YFP, n = 15; Chronos, n = 16) and time spent in the LED area per entry (YFP, n = 15; Chronos, n = 14) during the conditioning session. **g** Time spent in the LED area per entry during the retrieval session (YFP, n = 15; Chronos, n = 15). Time spent in the LED area per entry was calculated by excluding individuals that did not enter the LED area. Each circle represents results from one mouse. Data are represented as mean ± SEM. n.s., $p > 0.05$; $^{**}p < 0.01$ (Unpaired two-sided *t*-test).

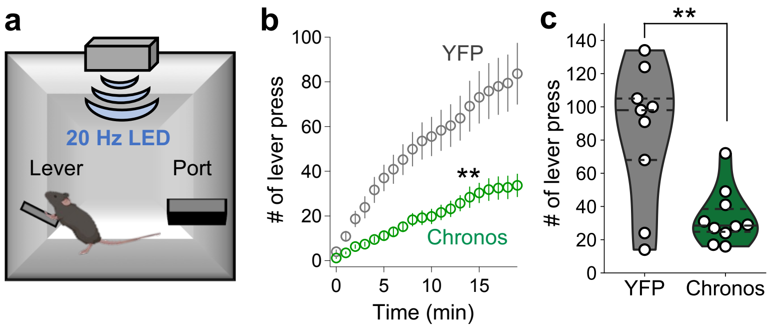

Supplementary Fig. 3 Photoactivation of the lPB-PSTN reduces the number of lever-press. a Schematic illustration of the lever-press task. **b** Time-series plots of the number of lever-press responses (YFP, n = 9; Chronos, n = 10). Data are represented as mean ± SEM. **p = 0.0012 (Two-way ANOVA). **c** Summary of the number of lever-press responses (YFP, n = 9; Chronos, n = 10). **p < 0.01 (Unpaired two-sided *t*-test).

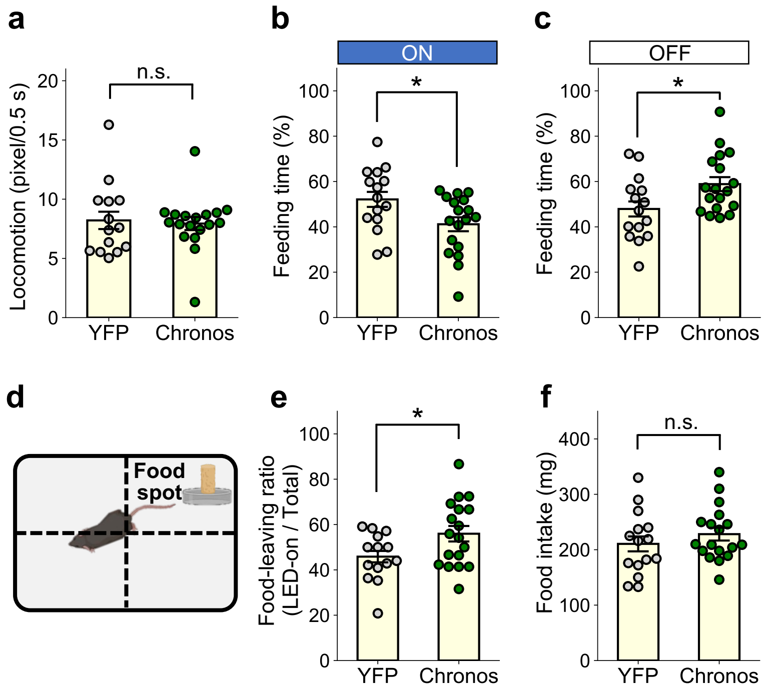

Supplementary Fig. 4 The lPB-PSTN stimulation suppresses feeding. **a** Locomotor activity during LED-on (6 min) (YFP, n = 14; Chronos, n = 18). **b, c** Ratios of time spent feeding for LED-on/Total (**b**) and ratios of time spent feeding for LED-off/Total (**c**) (YFP, n = 15; Chronos, n = 18). Each circle represents results from one mouse. **d** Schematic illustration of the food-leaving behavior. A quarter of the area was analyzed as a "food spot". Behavior involved in moving out of the food spot was defined as "food-leaving" behavior. **e** Ratio of food-leaving behavior during LED-on (6 min) to total food-leaving behavior (12 min) (YFP, n = 14; Chronos, n = 18). **f** Total food intake during the 15-min observation period (YFP, n = 15; Chronos, n = 18). Each circle represents each mouse. Data are represented as mean ± SEM. n.s., $p > 0.05$; *$p < 0.05$ (Unpaired two-sided *t*-test).

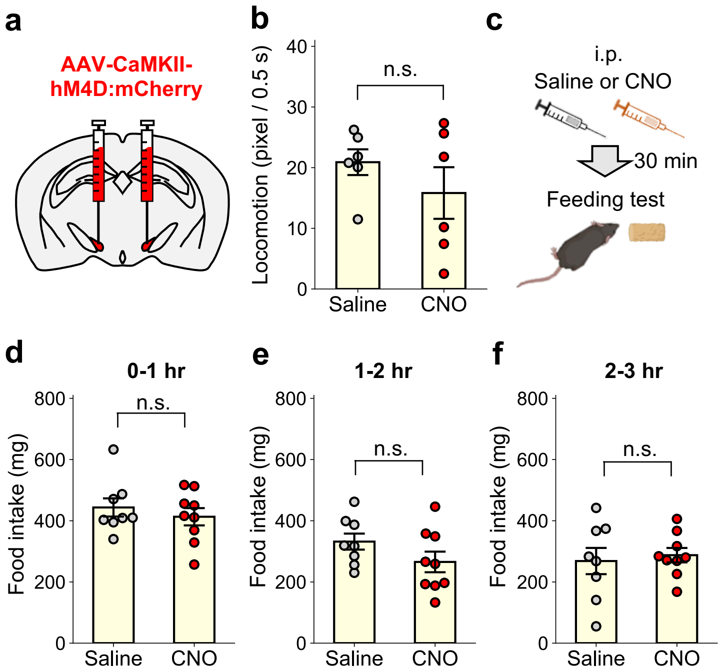

Supplementary Fig. 5 Effects of inhibition of the PSTN neurons on feeding behaviors. a Schematic of microinjection of AAV-CaMKII-hM4D:mCherry into the PSTN of the C57BL/6J mouse. **b** Locomotor activity during fear-induced suppression of feeding test (0–30 s) (Saline, n = 6; CNO, n = 6). **c** Schematic illustration of the feeding test to measure baseline food intake. **d–f** Comparison of food inake every an hour (Saline, n = 8; CNO, n = 9). Data are represented as mean ± SEM. n.s., $p > 0.05$ (Unpaired two-sided *t*-test).

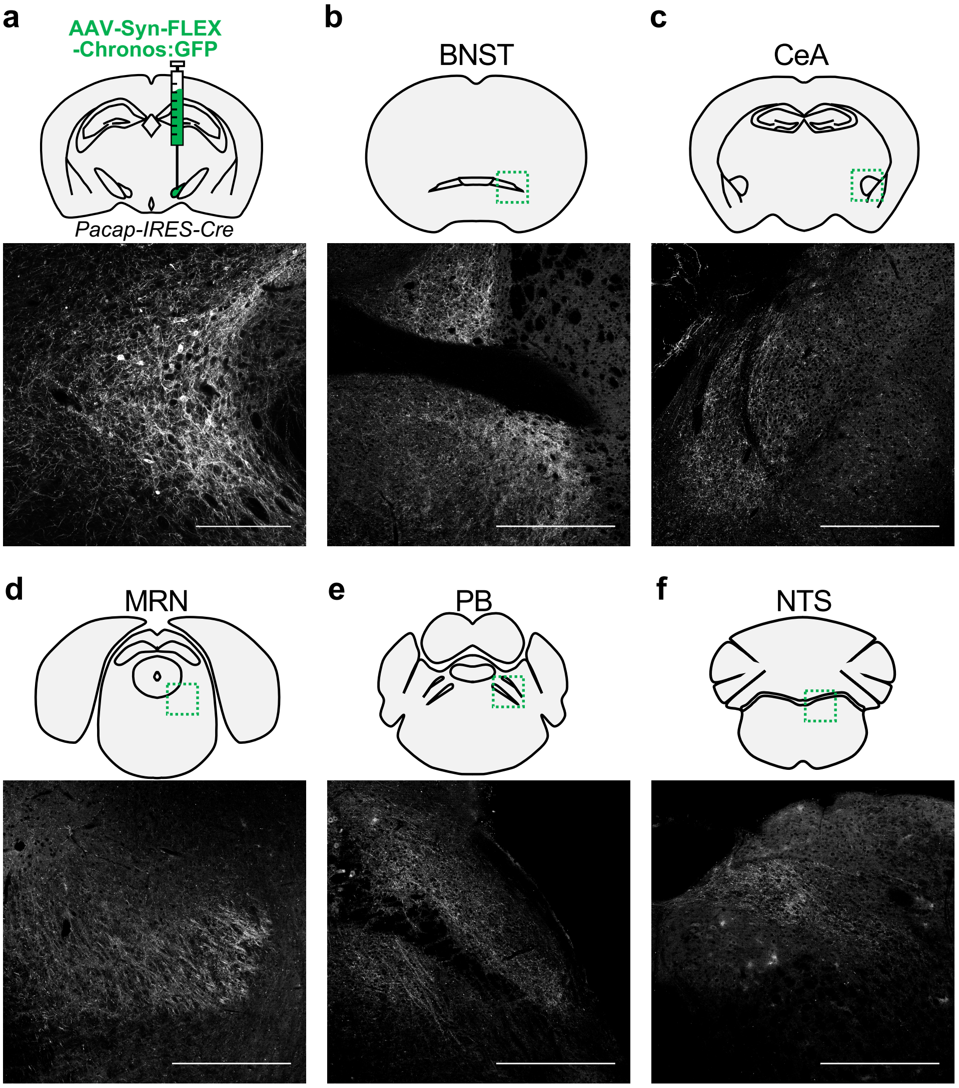

Supplementary Fig. 6 Projection of the PACAP^{PSTN} neurons. a–f Representative images of Chronos:GFP-labeled PACAP^{PSTN} neurons in the injection site (PSTN) (**a**) and the projection sites (**b–f**). Each scale bar represents 200 µm (**a**) or 500 µm (**b–f**). bed nucleus of stria terminalis (BNST), central amygdala (CeA), medial reticular nucleus (MRN), parabrachial nucleus (PB), and nucleus of the solitary tract (NTS). The brightness and contrast were adjusted to show each image optimally. Experiments were repeated independently in at least three mice with similar results.

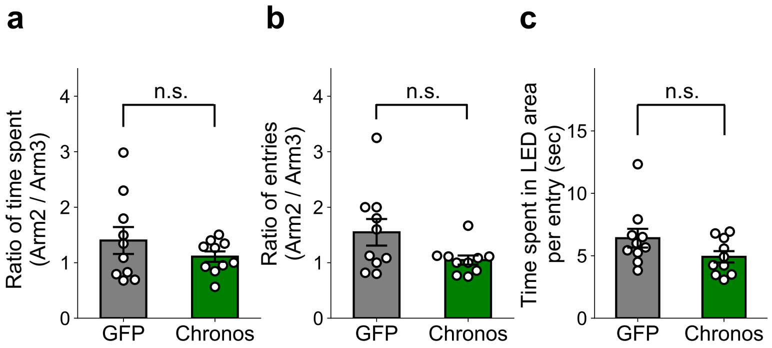

Supplementary Fig. 7 Real-time place avoidance of *Pacap-IRES-Cre* mice in the habituation session. a–c Summary of ratios of time spent in each arm, entries into each arm, and time spent in the LED area per entry during the habituation session. Each circle represents each mouse (GFP, n = 10; Chronos, n = 10). Data are represented as mean ± SEM. n.s., $p > 0.05$ (Unpaired two-sided *t*-test).

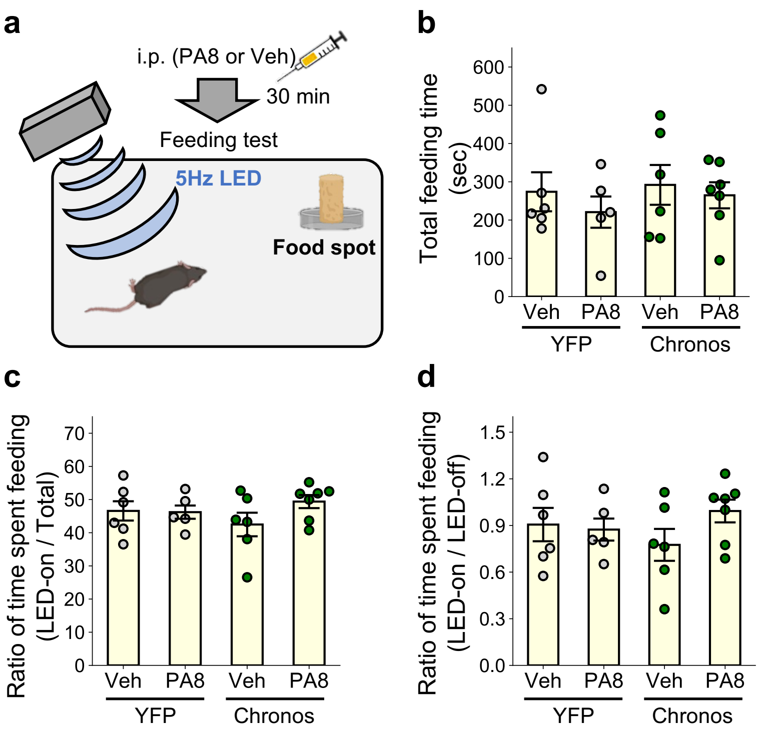

Supplementary Fig. 8 PA8 administration induces no acute effects on mouse feeding behavior. **a** Schematic illustration of feeding test. PAC1 antagonist PA8 or vehicle administration was performed 30 min before the start of the test. **b** Total feeding time during the 12-min observation period. **c** Ratio of time spent feeding for LED-on/Total. **d** Ratio of time spent feeding for LED-on/LED-off. Each circle represents results for one mouse (YFP-Veh, n = 6; YFP-PA8, n = 5; Chronos-Veh, n = 6; Chronos-PA8, n = 7). Data are represented as mean ± SEM. $P > 0.05$ (Two-way ANOVA).

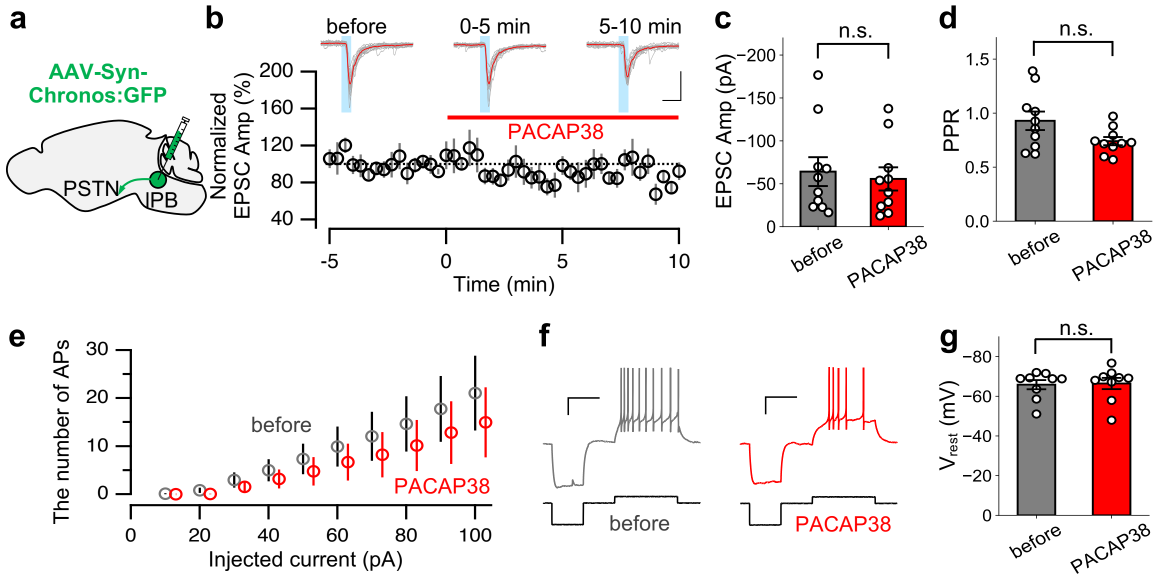

Supplementary Fig. 9 No strong effect of PACAP agonist on the lPB-PSTN pathway. a Bilateral injection of AAV-Syn-Chronos:GFP into C57BL/6J mice. **b** Representative traces of EPSCs (gray, 15 consecutive responses; red, average) evoked by photostimulation (every 20 s, 5-ms duration) (top). The bottom graph shows the timecourse of the effect of 10 nM PACAP38 on EPSC amplitude (n = 10). **c, d** Summary of the effects of PACAP38 on the EPSC amplitude and the paired-pulse ratio (PPR) (before, –5–0 min; PACAP38, 5–10 min, n = 10). **e** Summary of changes in the number of action potentials induced by step-current injection in PSTN neurons (n = 7). **f** Representative traces of firing patterns of the same cell before (gray) and during (red) PACAP38 application. **g** Summary of the resting membrane potentials (before, –5–0 min; PACAP38, 5-10 min, n = 9). Error bars indicate SEM. n.s., p > 0.05 (Paired two-sided *t*-test).

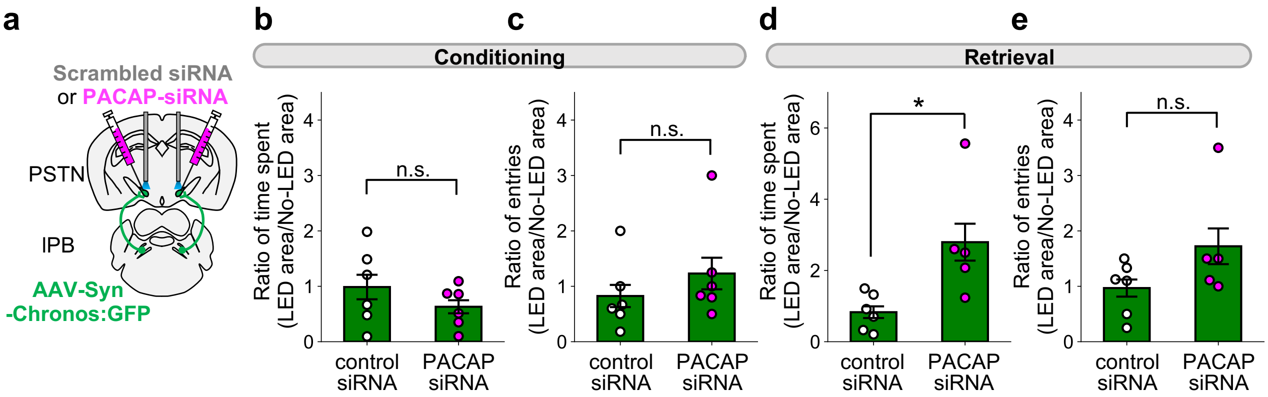

Supplementary Fig. 10 PACAP-siRNA administration attenuates aversive memory. a Schematic of microinjection of AAV-Syn-Chronos:GFP into the lPB and PACAP-siRNA or scrambled negative control siRNA into the PSTN of the C57BL/6J mouse. **b–e** The ratios of time spent and entries into each area (i.e., LED area/No LED area) during the latter half (5–10 min) of a conditioning session (scrambled siRNA, n = 6; PACAP-siRNA, n = 6) and the first half (0–5 min) of a retrieval session (scrambled siRNA, n = 6; PACAP-siRNA, n = 5). Each circle represents each mouse. Data are represented as mean ± SEM. n.s., $p > 0.05$; *$p < 0.05$ (Two-sided Mann-Whitney test).

Supplementary Table 1 Statistical summary

Fig. 2<br>Panel	Experiment	N	Test	t/F	P-values	Post-test	95% Cis	P-values
e	RTPA_conditioning_time spent_YFP	15	Two-way RM ANOVA, Bonferroni MC	Interaction: F (4, 112) = 1.525<br>Time: F (2.986, 83.60) = 0.2903<br>Arm: F (1, 28) = 2.118	P = 0.1998<br>P = 0.8313<br>P = 0.1567	Arm2 vs Arm3<br>0-2<br>2-4<br>4-6<br>6-8<br>8-10	-16.58 to 27.45<br>-7.503 to 32.44<br>-19.89 to 16.96<br>-1.771 to 34.90<br>-18.91 to 23.71	>0.9999<br>0.4697<br>>0.9999<br>0.0925<br>>0.9999
f	RTPA_conditioning_time spent_Chronos	16	Two-way RM ANOVA, Bonferroni MC	Interaction: F (4, 120) = 1.006<br>Time: F (3.458, 103.7) = 5.345<br>Arm: F (1, 30) = 9.329	P = 0.4075<br>P = 0.0047<br>P = 0.0011	Arm2 vs Arm3<br>0-2<br>2-4<br>4-6<br>6-8<br>8-10	-33.14 to 6.955<br>-45.68 to -0.2561<br>-43.87 to -0.8185<br>-44.31 to -5.257<br>-44.12 to 7.308	0.41<br>0.0466<br>0.0387<br>0.0091<br>0.2824
g	RTPA_conditioning_time spent	15× YFP, 16× Chronos	Unpaired t-test	t=2.759, df=17.20	P = 0.0133		-2.317 to -0.3098	
h	RTPA_conditioning_entry	15× YFP, 16× Chronos	Unpaired t-test	t=2.722, df=29	P = 0.0109		-1.468 to -0.2085	
k	RTPA_conditioning_moving speed	15× YFP, 16× Chronos	Unpaired t-test	t=3.833, df=19.52	P = 0.0011		8.065 to 27.39	
l	RTPA_conditioning_LED area	15× YFP, 16× Chronos	Unpaired t-test	t=4.327, df=29	P = 0.0002		-84.54 to -30.27	
m	RTPA_conditioning_correlation	15× YFP, 14× Chronos	linear regression (Pearson)	YFP: r = 0.1212<br>Chronos: r = -0.618	P = 0.6671<br>P = 0.0185		-0.4170 to 0.5964<br>-0.8650 to -0.1301	
n	RTPA_retrieval_time spent_YFP	15	Two-way RM ANOVA, Bonferroni MC	Interaction: F (4, 112) = 0.8077<br>Time: F (3.447, 96.50) = 1.238<br>Arm: F (1, 28) = 2.347	P = 0.5228<br>P = 0.3007<br>P = 0.1368	Arm2 vs Arm3<br>0-2<br>2-4<br>4-6<br>6-8<br>8-10	-11.87 to 27.61<br>-6.898 to 36.36<br>-13.18 to 23.31<br>-9.042 to 28.18<br>-17.24 to 21.30	>0.9999<br>0.3504<br>>0.9999<br>0.8184<br>>0.9999
o	RTPA_retrieval_time spent_Chronos	16	Two-way RM ANOVA, Bonferroni MC	Interaction: F (4, 120) = 3.400<br>Time: F (3.522, 105.6) = 0.3039<br>Arm: F (1, 30) = 5.434	P = 0.0113<br>P = 0.8525<br>P = 0.0267	Arm2 vs Arm3<br>0-2<br>2-4<br>4-6<br>6-8<br>8-10	-32.92 to -3.205<br>-22.70 to 7.766<br>-34.43 to 1.303<br>-18.30 to 12.30<br>-11.53 to 19.72	0.0114<br>0.9338<br>0.0803<br>>0.9999<br>>0.9999
p	RTPA_retrieval_time spent_0-5min	15× YFP, 16× Chronos	Unpaired t-test	t=3.341, df=20.54	P = 0.0032		-1.497 to -0.3474	
q	RTPA_retrieval_entry_0-5min	15× YFP, 16× Chronos	Unpaired t-test	t=2.464, df=18.86	P = 0.0235		-1.858 to -0.1508	
r	RTPA_retrieval_time spent_5-10min	15× YFP, 16× Chronos	Unpaired t-test	t=0.4941, df=29	P = 0.625		-0.7598 to 0.4641	
s	RTPA_retrieval_entry_5-10min	15× YFP, 16× Chronos	Unpaired t-test	t=0.8798, df=19.97	P = 0.3894		-0.5556 to 1.366	

Fig. 3<br>Panel	Experiment	N	Test	t/F	P-values	Post-test	95% Cis	P-values
h	Feeding_LED-on	15× YFP, 18× Chronos	Unpaired t-test	t=1.121, df=31	P = 0.2711		-44.05 to 12.81	
i	Feeding_LED-off	15× YFP, 18× Chronos	Unpaired t-test	t=1.683, df=31	P = 0.1025		-7.385 to 77.05	
j	Feeding_LED-on vs LED-off	15× YFP, 18× Chronos	Unpaired t-test	t=2.362, df=18.79	P = 0.0291		-0.9700 to -0.05819	
k	Feeding_total	15× YFP, 18× Chronos	Unpaired t-test	t=0.6393, df=31	P = 0.5274		-42.08 to 80.50	
l	Correlation_feeding vs avoidance	15× YFP, 16× Chronos	linear regression (Pearson)	YFP: r = -0.05796<br>Chronos: r = 0.5216	P = 0.8374<br>P = 0.0382		-0.5538 to 0.4682<br>0.03493 to 0.8083	

Fig. 4<br>Panel	Experiment	N	Test	t/F	P-values	Post-test	95% Cis	P-values
d	Food intake	6× saline, 6× CNO	Unpaired t-test	t=3.026, df=5.000	P = 0.0292		0.7028 to 8.631	
e	Total food intake	5× saline, 6× CNO	Unpaired t-test	t=2.481, df=9	P = 0.0349		23.44 to 508.6	

Fig. 5<br>Panel	Experiment	N	Test	t/F	P-values	Post-test	95% Cis	P-values
d	Food intake	4× GFP, 6× iChloC	Unpaired t-test	t=2.699, df=8	P = 0.0271		0.7043 to 8.962	
e	Total food intake	4× GFP, 6× iChloC	Unpaired t-test	t=4.367, df=8	P = 0.0024		89.95 to 291.2	

Fig. 7<br>Panel	Experiment	N	Test	t/F	P-values	Post-test	95% Cis	P-values
f	RTPA_conditioning_time spent	10× GFP, 10× Chronos	Unpaired t-test	t=2.502, df=18	P = 0.0222		-1.710 to -0.1490	
g	RTPA_conditioning_entry	10× GFP, 10× Chronos	Unpaired t-test	t=2.238, df=10.34	P = 0.0483		-2.117 to -0.009615	
h	RTPA_conditioning_time spent per entry	10× GFP, 10× Chronos	Unpaired t-test	t=1.741, df=18	P = 0.0987		-12.34 to 1.155	
j	RTPA_retrieval_time spent	10× GFP, 10× Chronos	Unpaired t-test	t=1.963, df=11.17	P = 0.075		-3.258 to 0.1832	
k	RTPA_retrieval_entry	10× GFP, 10× Chronos	Unpaired t-test	t=1.232, df=12.80	P = 0.2401		-2.187 to 0.6000	
l	RTPA_retrieval_time spent per entry	10× GFP, 9× Chronos	Unpaired t-test	t=2.803, df=17	P = 0.0122		-7.221 to -1.019	

Fig. 8<br>Panel	Experiment	N	Test	t/F	P-values	Post-test	95% Cis	P-values
g	Feeding_LED-on	10× GFP, 10× Chronos	Unpaired t-test	t=2.434, df=18	P = 0.0256		-107.9 to -7.924	
h	Feeding_LED-off	10× GFP, 10× Chronos	Unpaired t-test	t=1.624, df=18	P = 0.1219		-103.7 to 13.29	
i	Feeding_LED-on vs LED-off	10× GFP, 10× Chronos	Unpaired t-test	t=0.9280, df=18	P = 0.3657		-0.4186 to 0.1621	
j	Feeding_total	10× GFP, 10× Chronos	Unpaired t-test	t=2.252, df=18	P = 0.037		-199.3 to -6.918	
k	Correlation_feeding vs aversion	10× GFP, 10× Chronos	linear regression (Pearson)	YFP: r = 0.04659<br>Chronos: r = 0.752	P = 0.8983<br>P = 0.0121		-0.6007 to 0.6569<br>0.2324 to 0.9377	

Fig. 9<br>Panel	Experiment	N	Test	t/F	P-values	Post-test	95% Cis	P-values
c	RTPA_habituation_time spent	9× saline<br>9× CNO	Wilcoxon matched-pairs signed rank test<br>& Holm correction for MC<br>Saline (Arm2 vs Arm3)<br>CNO (Arm2 vs Arm3)		P = 0.7188<br>P = 0.8203			
d	RTPA_habituation_ratio of time spent	9× saline, 9× CNO	Wilcoxon matched-pairs signed rank test		P = 0.7344			
e	RTPA_conditioning_time spent	9× saline<br>9× CNO	Wilcoxon matched-pairs signed rank test<br>& Holm correction for MC<br>Saline (Arm2 vs Arm3)<br>CNO (Arm2 vs Arm3)		P = 0.1954<br>P = 0.3008			
f	RTPA_conditioning_ratio of time spent	9× saline, 9× CNO	Wilcoxon matched-pairs signed rank test		P = 0.0547			
g	RTPA_retrieval_time spent	9× saline<br>9× CNO	Wilcoxon matched-pairs signed rank test<br>& Holm correction for MC<br>Saline (Arm2 vs Arm3)<br>CNO (Arm2 vs Arm3)		P = 0.039<br>P = 0.3594			
h	RTPA_retrieval_ratio of time spent	9× saline, 9× CNO	Wilcoxon matched-pairs signed rank test		P = 0.0977			

Fig. 10<br>Panel	Experiment	N	Test	t/F	P-values	Post-test	95% Cis	P-values
c	RTPA_habituation_time spent	5× YFP-Veh<br>5× YFP-PA8<br>6× Chronos-Veh<br>7× Chronos-PA8	Paired t-test & Holm correction for MC<br>YFP-Veh (Arm2 vs Arm3)<br>YFP-PA8 (Arm2 vs Arm3)<br>Chronos-Veh (Arm2 vs Arm3)<br>Chronos-PA8 (Arm2 vs Arm3)		P = 0.7448<br>P = 0.8676<br>P = 0.7448<br>P = 0.9175			
d	RTPA_habituation_entry	5× YFP-Veh<br>5× YFP-PA8<br>6× Chronos-Veh<br>7× Chronos-PA8	Paired t-test & Holm correction for MC<br>YFP-Veh (Arm2 vs Arm3)<br>YFP-PA8 (Arm2 vs Arm3)<br>Chronos-Veh (Arm2 vs Arm3)<br>Chronos-PA8 (Arm2 vs Arm3)		P > 0.9999<br>P > 0.9999<br>P > 0.9999<br>P > 0.9999			
e	RTPA_conditioning_average speed	5× YFP-Veh<br>5× YFP-PA8	Unpaired t-test	t=0.2193, df=8	P = 0.8319		-0.3996 to 0.4836	
f	RTPA_conditioning_moving speed	6× Chronos-Veh<br>7× Chronos-PA8	Unpaired t-test	t=0.3105, df=11	P = 0.762		-24.78 to 18.65	
g	RTPA_conditioning_time spent	5× YFP-Veh<br>5× YFP-PA8<br>6× Chronos-Veh<br>7× Chronos-PA8	Paired t-test & Holm correction for MC<br>YFP-Veh (Arm2 vs Arm3)<br>YFP-PA8 (Arm2 vs Arm3)<br>Chronos-Veh (Arm2 vs Arm3)<br>Chronos-PA8 (Arm2 vs Arm3)		P = 0.6564<br>P = 0.6564<br>P = 0.0136<br>P = 0.0333			
h	RTPA_conditioning_entry	5× YFP-Veh<br>5× YFP-PA8<br>6× Chronos-Veh<br>7× Chronos-PA8	Paired t-test & Holm correction for MC<br>YFP-Veh (Arm2 vs Arm3)<br>YFP-PA8 (Arm2 vs Arm3)<br>Chronos-Veh (Arm2 vs Arm3)<br>Chronos-PA8 (Arm2 vs Arm3)		P > 0.9999<br>P > 0.9999<br>P = 0.0992<br>P = 0.261			
j	RTPA_retrieval_time spent	5× YFP-Veh<br>5× YFP-PA8<br>6× Chronos-Veh<br>7× Chronos-PA8	Paired t-test & Holm correction for MC<br>YFP-Veh (Arm2 vs Arm3)<br>YFP-PA8 (Arm2 vs Arm3)<br>Chronos-Veh (Arm2 vs Arm3)<br>Chronos-PA8 (Arm2 vs Arm3)		P > 0.9999<br>P > 0.9999<br>P = 0.008<br>P > 0.9999			
k	RTPA_retrieval_entry	5× YFP-Veh<br>5× YFP-PA8<br>6× Chronos-Veh<br>7× Chronos-PA8	Paired t-test & Holm correction for MC<br>YFP-Veh (Arm2 vs Arm3)<br>YFP-PA8 (Arm2 vs Arm3)<br>Chronos-Veh (Arm2 vs Arm3)<br>Chronos-PA8 (Arm2 vs Arm3)		P > 0.9999<br>P > 0.9999<br>P = 0.0116<br>P = 0.8907			

Supplementary Fig. 2<br>Panel	Experiment	N	Test	t/F	P-values	Post-test	95% Cis	P-values
b	RTPA_habituation_time spent	15× YFP, 16× Chronos	Unpaired t-test	t=1.379, df=29	P = 0.1784		-0.1249 to 0.6421	
c	RTPA_habituation_entry	15× YFP, 16× Chronos	Unpaired t-test	t=0.6291, df=29	P = 0.5342		-0.2009 to 0.3795	
d	RTPA_habituation_time spent per entry	15× YFP, 16× Chronos	Unpaired t-test	t=0.4580, df=29	P = 0.6504		-1.521 to 0.9647	
e	RTPA_conditioning_total distance	15× YFP, 16× Chronos	Unpaired t-test	t=0.5615, df=29	P = 0.5787		-3.879 to 2.208	
f	RTPA_conditioning_time spent per entry	15× YFP, 14× Chronos	Unpaired t-test	t=3.410, df=27	P = 0.0021		-6.031 to -1.500	
g	RTPA_retrieval_time spent per entry	15× YFP, 15× Chronos	Unpaired t-test	t=1.986, df=28	P = 0.0569		-2.988 to 0.04608	

Supplementary Fig. 3<br>Panel	Experiment	N	Test	t/F	P-values	Post-test	95% Cis	P-values
b	lever-press	9× YFP, 10× Chronos	Two-way ANOVA	Interaction: F (19, 323) = 8.688<br>Time: F (19, 323) = 56.62<br>AAV: F (1, 17) = 14.92	P < 0.0001<br>P < 0.0001<br>P = 0.0012		14.26 to 48.59	
c	lever-press	9× YFP, 10× Chronos	Unpaired t-test	t=3.427, df=10.34	P = 0.0062		-83.72 to -17.92	

Supplementary Fig. 4<br>Panel	Experiment	N	Test	t/F	P-values	Post-test	95% Cis	P-values
a	Locomotion	14× YFP, 18× Chronos	Unpaired t-test	t=0.2770, df=30	P = 0.7836		-2.233 to 1.700	
b	Feeding_LED-on (%)	15× YFP, 18× Chronos	Unpaired t-test	t=2.318, df=31	P = 0.0272		-20.60 to -1.318	
c	Feeding_LED-off (%)	15× YFP, 18× Chronos	Unpaired t-test	t=2.318, df=31	P = 0.0272		1.318 to 20.60	
e	Food-leaving	14× YFP, 18× Chronos	Unpaired t-test	t=2.188, df=30	P = 0.0366		0.6754 to 19.54	
f	Food intake	15× YFP, 18× Chronos	Unpaired t-test	t=0.9517, df=31	P = 0.3486		-20.09 to 55.25	

Supplementary Fig. 5<br>Panel	Experiment	N	Test	t/F	P-values	Post-test	95% Cis	P-values
b	Locomotion	6× saline, 6× CNO	Unpaired t-test	t=1.069, df=10	P = 0.3104		-15.68 to 5.515	
d	Food intake_0-1h	8× saline, 9× cno	Unpaired t-test	t=0.7207, df=15	P = 0.4822		-120.3 to 59.48	
e	Food intake_1-2h	8× saline, 9× cno	Unpaired t-test	t=1.498, df=15	P = 0.1548		-160.9 to 28.06	
f	Food intake_2-3h	8× saline, 9× cno	Unpaired t-test	t=0.3875, df=15	P = 0.7038		-86.31 to 124.7	

Supplementary Fig. 7<br>Panel	Experiment	N	Test	t/F	P-values	Post-test	95% Cis	P-values
a	RTPA_habituation_time spent	10× GFP, 10× Chronos	Unpaired t-test	t=1.116, df=11.62	P = 0.2871		-0.8589 to 0.2785	
b	RTPA_habituation_entry	10× GFP, 10× Chronos	Unpaired t-test	t=1.977, df=11.08	P = 0.0735		-1.057 to 0.05622	
c	RTPA habituation, staying time per entry	10× GFP, 10× Chronos	Unpaired t-test	t=1.697, df=18	P = 0.1069		-3.338 to 0.3547	

Supplementary Fig. 8<br>Panel	Experiment	N	Test	t/F	P-values	Post-test	95% Cis	P-values
b	Feeding_total	6× YFP-Veh<br>5× YFP-PA8<br>6× Chronos-Veh<br>7× Chronos-PA8	Two-way ANOVA	Interaction: F (1, 20) = 0.07122<br>IP: F (1, 20) = 0.6806<br>AAV: F (1, 20) = 0.4047	P = 0.7923<br>P = 0.4191<br>P = 0.5319			
c	Feeding_ LED-on vs total	6× YFP-Veh<br>5× YFP-PA8<br>6× Chronos-Veh<br>7× Chronos-PA8	Two-way ANOVA	Interaction: F (1, 20) = 1.548<br>IP: F (1, 20) = 1.248<br>AAV: F (1, 20) = 0.02417	P = 0.2278<br>P = 0.2772<br>P = 0.8780			
d	Feeding_LED-on vs LED-off	6× YFP-Veh<br>5× YFP-PA8<br>6× Chronos-Veh<br>7× Chronos-PA8	Two-way ANOVA	Interaction: F (1, 20) = 1.626<br>IP: F (1, 20) = 0.8964<br>AAV: F (1, 20) = 0.003261	P = 0.2169<br>P = 0.3551<br>P = 0.9550			

Supplementary Fig. 9<br>Panel	Experiment	N	Test	t/F	P-values	Post-test	95% Cis	P-values
c	EPSC Amp	10 neurons, 7 mice	Paired t-test	t=1.513, df=9	P = 0.1646		-4.210 to 21.22	
d	PPR	10 neurons, 7 mice	Paired t-test	t=2.179, df=9	P = 0.0573		-0.3834 to 0.007180	
g	V rest	9 neurons, 7 mice	Paired t-test	t=0.2170, df=8	P = 0.8337		-6.873 to 5.691	

Supplementary Fig. 10<br>Panel	Experiment	N	Test	t/F	P-values	Post-test	95% Cis	P-values
b	RTPA_conditioning_time spent	6× control, 6× PACAP	Mann Whitney test		P = 0.4848			
c	RTPA_conditioning_entry	6× control, 6× PACAP	Mann Whitney test		P = 0.29			
d	RTPA_retrieval_time spent	6× control, 5× PACAP	Mann Whitney test		P = 0.0173			
e	RTPA_retrieval_entry	6× control, 5× PACAP	Mann Whitney test		P = 0.2403			
