# Supplementary material for: Parabrachial-to-parasubthalamic nucleus pathway mediates fear-induced suppression of feeding in male mice
Source: Nat Commun. 2022 Dec 30;13:7913. doi: 10.1038/s41467-022-35634-2 (PMC9803671; doi:10.1038/s41467-022-35634-2)
Supplement: Supplementary file 2 — Description of Additional Supplementary Files [file 41467_2022_35634_MOESM2_ESM.pdf]

Description of Additional Supplementary Files

File Name: Supplementary Data 1
Description: **Whole-brain projection patterns of lPB neurons.** The movie shows neuronal projections in a C57BL/6J mouse brain injected with AAV-Syn-Chronos:GFP into the bilateral lPB.

File Name: Supplementary Data 2
Description: **YFP-control and Chronos mice in the real-time place avoidance.** The movie shows a representative conditioning session for the real-time place avoidance of C57BL/6J mice injected with AAV-Syn-YFP or AAV-Syn-Chronos:GFP into the lPB. The movie contains behavior during the latter half (5–10 min) of the conditioning session and is displayed at 10× speed. Mice received 5 ms of LED illumination (40 Hz) whenever they were present in the LED area (blue circle).

File Name: Supplementary Data 3
Description: **Chronos mouse in the feeding test.** The movie shows a representative 12-min feeding test of a C57BL/6J mouse injected with AAV-Syn-Chronos:GFP into the lPB. The mouse was allowed access to a food pellet in a test chamber. During LED-on periods, the mouse was received 5 ms of LED illumination (five 5-Hz pulses with a 5-s inter-train-interval).
