# Supplementary material for: Parabrachial-to-parasubthalamic nucleus pathway mediates fear-induced suppression of feeding in male mice
Source: Nat Commun. 2022 Dec 30;13:7913. doi: 10.1038/s41467-022-35634-2 (PMC9803671; doi:10.1038/s41467-022-35634-2)
Supplement: Supplementary file 6 — Reporting Summary [file 41467_2022_35634_MOESM6_ESM.pdf]

nature portfolio

Corresponding author(s): Ayako M. Watabe

Last updated by author(s): 2022/11/23

Reporting Summary

Nature Portfolio wishes to improve the reproducibility of the work that we publish. This form provides structure for consistency and transparency in reporting. For further information on Nature Portfolio policies, see our Editorial Policies and the Editorial Policy Checklist.

Statistics

For all statistical analyses, confirm that the following items are present in the figure legend, table legend, main text, or Methods section.

n/a	Confirmed	
☐	☒	The exact sample size (*n*) for each experimental group/condition, given as a discrete number and unit of measurement
☐	☒	A statement on whether measurements were taken from distinct samples or whether the same sample was measured repeatedly
☐	☒	The statistical test(s) used AND whether they are one- or two-sided<br>*Only common tests should be described solely by name; describe more complex techniques in the Methods section.*
☐	☒	A description of all covariates tested
☐	☒	A description of any assumptions or corrections, such as tests of normality and adjustment for multiple comparisons
☐	☒	A full description of the statistical parameters including central tendency (e.g. means) or other basic estimates (e.g. regression coefficient) AND variation (e.g. standard deviation) or associated estimates of uncertainty (e.g. confidence intervals)
☐	☒	For null hypothesis testing, the test statistic (e.g. *F*, *t*, *r*) with confidence intervals, effect sizes, degrees of freedom and *P* value noted<br>*Give P values as exact values whenever suitable.*
☒	☐	For Bayesian analysis, information on the choice of priors and Markov chain Monte Carlo settings
☐	☒	For hierarchical and complex designs, identification of the appropriate level for tests and full reporting of outcomes
☐	☒	Estimates of effect sizes (e.g. Cohen's *d*, Pearson's *r*), indicating how they were calculated

Our web collection on statistics for biologists contains articles on many of the points above.

Software and code

Policy information about availability of computer code

Data collection	Real-time place aversion data were collected through Time OFCR1 software (O'Hara & Co., Ltd.). Feeding data were collected using TimeFZ software (O'HARA & CO., Ltd.), a web camera (HD Webcam C525, Logicool), and its software (version 2.51, Logicool). Lever-press data were obtained using Operant Task Studio V2 (O'HARA & CO., Ltd.). Electrophysiological data in slices was collected using LabChart 7 (AD Instruments). Whole brain images were acquired through FAST system. Images of immunohistochemistry and axonal projection mapping were acquired using a confocal microscope (FV3000, Olympus).
Data analysis	Statistics was performed by GraphPad Prism 9 (GraphPad Software). Electrophysiological data in slices was analyzed using Igor Pro 7 (WaveMetrics). The fluorescent images were analyzed by ImageJ Fiji (version 1.0).

For manuscripts utilizing custom algorithms or software that are central to the research but not yet described in published literature, software must be made available to editors and reviewers. We strongly encourage code deposition in a community repository (e.g. GitHub). See the Nature Portfolio guidelines for submitting code & software for further information.

Data

Policy information about availability of data

All manuscripts must include a data availability statement. This statement should provide the following information, where applicable:

- Accession codes, unique identifiers, or web links for publicly available datasets
- A description of any restrictions on data availability
- For clinical datasets or third party data, please ensure that the statement adheres to our policy

The data that support the findings in this study are available from the authors upon reasonable request.

Human research participants

Policy information about studies involving human research participants and Sex and Gender in Research.

Reporting on sex and gender	*Use the terms sex (biological attribute) and gender (shaped by social and cultural circumstances) carefully in order to avoid confusing both terms. Indicate if findings apply to only one sex or gender; describe whether sex and gender were considered in study design whether sex and/or gender was determined based on self-reporting or assigned and methods used. Provide in the source data disaggregated sex and gender data where this information has been collected, and consent has been obtained for sharing of individual-level data; provide overall numbers in this Reporting Summary. Please state if this information has not been collected. Report sex- and gender-based analyses where performed, justify reasons for lack of sex- and gender-based analysis.*
Population characteristics	*Describe the covariate-relevant population characteristics of the human research participants (e.g. age, genotypic information, past and current diagnosis and treatment categories). If you filled out the behavioural & social sciences study design questions and have nothing to add here, write "See above."*
Recruitment	*Describe how participants were recruited. Outline any potential self-selection bias or other biases that may be present and how these are likely to impact results.*
Ethics oversight	*Identify the organization(s) that approved the study protocol.*

Note that full information on the approval of the study protocol must also be provided in the manuscript.

Field-specific reporting

Please select the one below that is the best fit for your research. If you are not sure, read the appropriate sections before making your selection.

☒ Life sciences ☐ Behavioural & social sciences ☐ Ecological, evolutionary & environmental sciences

For a reference copy of the document with all sections, see nature.com/documents/nr-reporting-summary-flat.pdf

Life sciences study design

All studies must disclose on these points even when the disclosure is negative.

Sample size	No statistical methods were used to pre-determine sample sizes, but our sample sizes were selected based on related research.<br>Bowen et al., 2020, eLife (doi: 10.7554/eLife.59799.)<br>Ito et al., 2021, Mol Brain (doi: 10.1186/s13041-021-00807-5.)
Data exclusions	One or two samples for which a value (time spent per entry) cannot be calculated were excluded. One specimen in schematic illustration of the LED cannula unit was excluded because of the indistinct tip. One sample that contained non-typical EPSC was excluded in electrophysiological data.
Replication	All experiments were replicated at least once, and similar results were obtained.
Randomization	Animals were randomly allocated to the different experimental conditions reported in this study.
Blinding	Blinding was used for data collection and analysis in the behavioral experiments.

Reporting for specific materials, systems and methods

We require information from authors about some types of materials, experimental systems and methods used in many studies. Here, indicate whether each material, system or method listed is relevant to your study. If you are not sure if a list item applies to your research, read the appropriate section before selecting a response.

Materials & experimental systems

n/a	Involved in the study
☐	☒ Antibodies
☒	☐ Eukaryotic cell lines
☒	☐ Palaeontology and archaeology
☐	☒ Animals and other organisms
☒	☐ Clinical data
☒	☐ Dual use research of concern

Methods

n/a	Involved in the study
☒	☐ ChIP-seq
☒	☐ Flow cytometry
☒	☐ MRI-based neuroimaging

Antibodies

Antibodies used	We described information about antibodies in the method section.<br>Rabbit polyclonal antibody against PACAP-38 (1: 400, BMA, Rheinstrasse, Switzerland; T-4473)<br>Mouse monoclonal antibody (SP-DE4-21) against SubstanceP/Tac1 (1: 200, abcam, Cambridge, UK, ab14184)<br>Guinea Pig polyclonal antibody against CRF (1:300, BMA, Rheinstrasse, Switzerland; T-5007)<br>Goat anti-rabbit biotinylated antibody (1:200, Newark, USA;BA-1000-1.5)<br>Goat anti mouse Alexa488 (1:200, Thermo Fisher Scientific, MA USA; A-11029)<br>Goat anti gunia pig Alexa647 (1:200, Thermo Fisher Scientific, MA USA; A-21450)<br>Streptavidin Alexa594 (1:500, Thermo Fisher Scientific, MA USA; S11227)
Validation	The specificity of all primary antibodies were validated from each manufacture sites or previous studies.<br>https://www.bma.ch/files/product/t-4473.pdf<br>https://www.abcam.com/substance-p-antibody-sp-de4-21-ab14184.html<br>Chee et al., 2013, J Comp Neurol (doi:10.1002/cne.23273.)

Animals and other research organisms

Policy information about studies involving animals; ARRIVE guidelines recommended for reporting animal research, and Sex and Gender in Research

Laboratory animals	C57BL/6J mice and Pacap-IRES-Cre mice (a gift from Prof. Bradford B. Lowell, Harverd University) were group-housed in temperature (20–24°C) and humidity (45–65%) controlled environments on a 12 h light/dark cycle. Mice were used for behavioral tests, immunohistochemistry, and electrophysiology.
Wild animals	This study did not involve wild animals.
Reporting on sex	Data in main figures were collected in male mice. The histological analysis was performed in female mice (Supplementary Fig.6). The sex was described in title, abstract, and method.
Field-collected samples	This study did not involve samples collected from the field.
Ethics oversight	The care and use of experimental animals and approved by the Institutional Animal Care and Use Committee of the Jikei University (Approval Number 2018-030, 2019-010) and conformed to the Guidelines for the Proper Conduct of Animal Experiments of the Science Council of Japan (2006).

Note that full information on the approval of the study protocol must also be provided in the manuscript.
